# Exposure-duration-associated auditory dysfunction and cochlear presynaptic ribbon alterations during simulated high-altitude hypobaric hypoxia in mice

**DOI:** 10.3389/fneur.2026.1963563

**Published:** 2026-10-15

**Authors:** Qingping Zhang, Guanghao Yue, Ying Zhang, Shanhong Li, Wenjun Cao, Xiaoli Zhang, Benhong Ren, Chenguang Zhang, Chaohua Wang, Wanru Chen, Tana Wuren, Bin Guo, Yi Wang

**Affiliations:** 1 Department of Otolaryngology-Head and Neck Surgery, Qinghai University Affiliated Hospital (The Clinical Medical School), Qinghai University, Xining, China; 2 Research Center for High Altitude Medicine, School of Medicine, Qinghai University, Xining, China; 3 Key Laboratory for Application of High-altitude Medicine, Qinghai University, Xining, China; 4 Department of Otolaryngology, Peking Union Medical College Hospital, Chinese Academy of Medical Sciences and Peking Union Medical College, Beijing, China

**Keywords:** auditory brainstem response, cochlea, CtBP2-positive presynaptic ribbons, high altitude, hypobaric hypoxia, mitochondrial dysfunction, oxidative stress

## Abstract

**Background:**

High-altitude hypobaric hypoxia can alter auditory function, but the temporal relationships among cochlear dysfunction, hair-cell integrity, inner-hair-cell presynaptic ribbons, and redox-bioenergetic disturbances remain unclear.

**Methods:**

Eighty male C57BL/6J mice were assigned to an ambient-pressure control group in Xining, China (approximately 2,260 m), or to nominal 3-, 15-, or 30-day simulated hypobaric-hypoxia exposure groups (*n* = 20 per group). Chamber entry was staggered, and terminal assessments and tissue collection were completed within a period of less than 24 h. Separate animal subsets were used for auditory brainstem response (ABR) recording and hematological assessment. ABR thresholds, wave I latency, and wave I peak-to-trough amplitude were evaluated. Hair-cell survival and CtBP2-positive presynaptic ribbons were assessed by confocal microscopy. Hematological indices, 4-hydroxynonenal (4-HNE) and 8-hydroxy-2′-deoxyguanosine-related immunoreactivity, whole-cochlear ATP content, and JC-1-defined mitochondrial polarization were also assessed.

**Results:**

Red blood cell count, hemoglobin concentration, and hematocrit were increased at all exposure durations. Exposure group was associated with altered ABR thresholds and wave I measures. Control-focused comparisons showed prolonged wave I latency at 4 and 8 kHz in all hypoxia groups, whereas wave I amplitude at 90 dB SPL was lower at both frequencies only after 15 days. OHC survival did not differ significantly among groups, and no overt IHC loss was observed. CtBP2-positive puncta were reduced in the basal turn after 3 days and in all cochlear turns after 15 days. 4-HNE immunoreactivity increased at all time points, whereas 8-OHdG-related immunoreactivity increased after 3 and 15 days. Whole-cochlear ATP content was reduced at all time points. At 30 days, JC-1 analysis indicated a shift toward mitochondrial depolarization in mixed cochlear-cell suspensions.

**Conclusion:**

Simulated high-altitude hypobaric-chamber exposure was associated with an exposure-duration-associated and endpoint-specific cochlear phenotype. Auditory abnormalities and presynaptic ribbon alterations occurred without substantial hair-cell loss and were accompanied by oxidative and bioenergetic disturbances. The pattern of abnormalities varied across exposure groups and endpoints.

## 1 Introduction

High-altitude environments are characterized by reduced barometric pressure and inspired oxygen partial pressure, resulting in systemic hypobaric hypoxia. Human studies have reported changes in hearing thresholds, otoacoustic emissions, and auditory brainstem responses (ABRs) during acute or prolonged altitude exposure, although the findings vary with altitude, exposure duration, acclimatization, and test conditions (1, 2). Animal studies further suggest that high-altitude hypoxia can cause time-dependent auditory dysfunction and structural abnormalities involving cochlear hair cells and spiral ganglion neurons (3, 4). A recent time-series study by our group in C57BL/6J mice documented dynamic changes in ABR, electrocochleography, and otoacoustic emissions during simulated hypobaric hypoxia (5). However, the cochlear cellular substrates underlying these functional abnormalities remain insufficiently defined. The cochlea has high metabolic demands and depends on tightly regulated microcirculation to maintain sensory transduction (6). Outer hair cells provide cochlear amplification, whereas inner hair cells transmit acoustic information to auditory nerve fibers through specialized ribbon synapses (7). Synaptic ribbons support rapid and temporally precise neurotransmitter release, and ribbon synaptopathy can precede overt hair-cell loss or permanent threshold elevation (8–10). ABR wave I predominantly reflects synchronized auditory nerve output, whereas CtBP2-positive puncta provide a structural estimate of presynaptic ribbons in inner hair cells (10, 11). Because neither endpoint alone establishes the number of intact functional synapses, integrating ABR wave I with CtBP2 quantification may provide a more informative structure–function assessment than threshold measurement alone.

Mitochondrial oxidative stress is a biologically plausible link between hypobaric hypoxia and cochlear injury. Hypoxia alters mitochondrial electron transport and redox signaling, and sustained reactive oxygen species (ROS) accumulation can impair energy metabolism and promote oxidative damage (12, 13). Oxidative stress and mitochondrial dysfunction are implicated in multiple forms of acquired sensorineural hearing loss (13). Experimentally increased ROS can also reduce inner-hair-cell ribbon-associated structures before extensive hair-cell degeneration. Building on prior physiological time-course work (5), the present study integrates ABR thresholds and wave I measures with hair-cell morphology, CtBP2-positive presynaptic ribbons, oxidative-damage-associated immunoreactivity, cellular energy status, and mitochondrial polarization.

In the present study, mice were exposed to simulated hypobaric hypoxia equivalent to approximately 5,500 m for 3, 15, or 30 days. Auditory function was evaluated using ABR thresholds together with wave I amplitude and latency, whereas cochlear injury was assessed by examining IHC and OHC integrity and CtBP2-positive presynaptic ribbons. Cochlear 4-HNE and 8-OHdG-related immunoreactivity, ATP content, and mitochondrial membrane potential were assessed to characterize lipid-peroxidation-associated stress, oxidized-guanine-related damage, cellular energy status, and mitochondrial polarization. We aimed to define the exposure-duration-associated relationships among auditory dysfunction, hair-cell and presynaptic-ribbon pathology, and redox–bioenergetic disturbance. We hypothesized that auditory-nerve function and IHC presynaptic ribbons would be altered in the absence of extensive hair-cell loss and that these abnormalities would be accompanied by oxidative and energetic stress.

## 2 Materials and methods

### 2.1 Animals, ethics, and experimental design

All animal procedures were approved by the Experimental Animal Ethics Committee of Qinghai University Affiliated Hospital (approval no. P-SL-2024181; approved on December 28, 2024) and were conducted in accordance with institutional and national guidelines for the care and use of laboratory animals. The study is reported in accordance with the ARRIVE 2.0 guidelines, where applicable. Male C57BL/6J mice, 4 weeks of age and weighing 20–24 g, were obtained from SPF (Beijing) Biotechnology Co., Ltd. (Beijing, China) and housed at the Research Center for High Altitude Medicine, School of Medicine, Qinghai University. After an acclimatization period of 1 week, mice underwent otoscopic examination. Animals with cerumen impaction, otitis externa or media, tympanic-membrane perforation, or another external or middle-ear abnormality were excluded before group allocation. The formal experiment included 80 male C57BL/6J mice assigned to four groups (*n* = 20 per group): an ambient-pressure control group and nominal 3-, 15-, and 30-day hypobaric-hypoxia exposure groups. Chamber entry was staggered, with entry on 2 June, 21 May, and 6 May 2026, respectively, and terminal assessments and tissue collection were completed within a period of less than 24 h on 5–6 June 2026. The staggered exposure schedule began after acclimatization, at approximately 5 weeks of age; individual groups entered the chamber at different ages. Exposure ended when each mouse was removed from the chamber; the duration labels denote planned exposure groups. No *a priori* sample-size calculation was performed. The sample size for cochlear whole-mount analyses (three mice per group) was selected empirically during study planning. Within each group, 10 mice underwent terminal blood collection without prior ABR testing, and a separate 10 underwent left-ear ABR recording. In the blood-collection subgroup, bilateral cochleae from five mice were pooled within each animal for ATP measurement, and bilateral cochleae from three different mice were pooled within each animal for JC-1 analysis. Both cochleae from the remaining two mice were stored at −80 °C. At tissue collection, three mice per group were randomly selected from the ABR subgroup for cochlear whole-mount analysis, and five different ABR-tested mice provided cryosections for 4-HNE and 8-OHdG-related immunostaining. One ear was randomly selected for histology in each of these eight mice, and the contralateral cochlea was stored at −80 °C. Both cochleae from the remaining two ABR-tested mice were also stored at −80 °C. Thus, 16 distinct mice per group provided tissue for the reported cochlear assays, while all 20 contributed ABR or hematological measurements. In total, 16 cochleae from 12 mice per group were stored for future studies. The sample size for each outcome is also reported in its figure legend. The allocation of animals and cochleae to terminal assessments and tissue assays is summarized in Supplementary Table S2.

### 2.2 Hypobaric hypoxia exposure, animal monitoring, randomization, and blinding

Hypobaric hypoxia was generated in a low-pressure chamber set to simulate an altitude of approximately 5,500 m. Chamber conditions were continuously monitored during exposure. Representative steady-state values recorded during chamber operation were as follows: simulated altitude, 5,502 m; barometric pressure, 49.35 kPa; oxygen fraction, 19.1%; oxygen partial pressure, 9.43 kPa; normobaric-equivalent oxygen concentration at sea level, 9.89%; temperature, 20.7 °C; relative humidity, 45.1%; carbon dioxide concentration, 3,162 ppm; and ventilation rate, 107 m^3^/h. The chamber was decompressed from local ambient pressure to the target altitude over 40 min and was recompressed to ambient pressure over 30 min. During terminal ABR assessments, the main chamber remained at the target hypobaric conditions, and animals were transferred out in pairs through the chamber transfer port. Control mice were housed outside the hypobaric chamber in the animal facility of the Research Center for High Altitude Medicine, School of Medicine, Qinghai University, under local ambient-pressure conditions in Xining, China, at approximately 2,260 m above sea level. No chamber-matched ambient-pressure control group was included. Because the control animals were not maintained at sea level, they are referred to throughout the manuscript as ambient-pressure controls rather than sea-level normoxic controls. All mice were maintained on a 12-h light/dark cycle at 22 ± 2 °C and 50 ± 10% relative humidity, with no more than five animals per cage and ad libitum access to food and water. Animals were monitored at least once daily during acclimatization and exposure for general appearance, spontaneous activity, grooming, respiration, body condition, and access to food and water. Humane endpoints included severe respiratory distress, inability to access food or water, moribund appearance, or marked weight loss in accordance with institutional guidance. Group allocation was performed by simple randomization documented in the laboratory record. Blinding was not feasible during chamber exposure. Physiological recordings were performed with knowledge of group assignment; however, histological image quantification and the primary statistical analyses were performed using coded files by an assessor unaware of group identity. Cage position and the order of tissue processing, staining, imaging, and image analysis were alternated as far as practicable. The order of ABR testing is described in Section 2.4.

### 2.3 Hematological assessment

Hematological indices were measured in 10 mice per group that had not undergone ABR recording. These mice were euthanized by cervical dislocation, followed immediately by decapitation. No hematological measurements were obtained from the separate ABR subgroup. Approximately 200 μL of trunk blood was collected directly into a 1.5-mL EDTA-anticoagulated polypropylene microcentrifuge tube (HR10013; Nantong Hairui Laboratory Equipment Co., Ltd., Nantong, China), followed immediately by cochlear harvesting. Blood analysis and cochlear sample processing proceeded in parallel; tissue collection did not await completion of hematological analysis. Red blood cell count (RBC), hemoglobin concentration (HGB), and hematocrit (HCT) were measured using a BC-5000 Vet automated veterinary hematology analyzer (Shenzhen Mindray Bio-Medical Electronics Co., Ltd., Shenzhen, China) with manufacturer-matched veterinary hematology reagents. Samples were analyzed on the day of collection.

### 2.4 Auditory brainstem response recordings

Auditory brainstem responses (ABRs) were recorded in *n* = 10 mice per group using a Neuro-Audio auditory evoked-potential system (Youting, Guangzhou, China) in a sound-attenuating and electrically shielded booth with background noise <25 dB. ABR recordings were obtained only at the terminal assessment; pre-exposure baseline ABR measurements were not collected. Anesthesia was induced in an induction chamber using 2.5% isoflurane in oxygen at a flow rate of 2.0 L/min and was maintained via a nose cone using 1.5% isoflurane in oxygen at 0.5 L/min. Anesthetic depth was assessed periodically by the absence of the pedal withdrawal reflex and by monitoring respiratory pattern, and the vaporizer setting was adjusted when necessary. Body temperature was maintained at 37.0 ± 0.5 °C using a thermostatically controlled heating pad. ABR recordings from all 40 mice were obtained sequentially using a single recording system within the terminal-assessment period of less than 24 h. For each of the nominal 3-, 15-, and 30-day hypobaric-exposure groups, mice were transferred through a small chamber transfer port in batches of two while the main chamber remained at the target hypobaric conditions. Mice that had not yet been transferred remained inside the chamber. To minimize the duration of reoxygenation after removal from the hypobaric chamber, ABRs were recorded from the left ear only. Within each group, mice were randomly selected for testing, and all animals in one group were tested before proceeding to the next group. While the first mouse in a pair underwent ABR recording, the second remained awake without anesthesia or other preparation. Induction of anesthesia took approximately 2–3 min, and left-ear ABR recording took approximately 20 min per mouse. Each mouse was euthanized after completion of ABR recording, and the next pair was transferred after both mice in the preceding pair had completed testing and euthanasia. Ambient-pressure controls did not undergo chamber transfer. A soft rubber acoustic tube was gently inserted into the left external auditory canal for stimulus delivery. Recordings were initiated as soon as practicable after chamber removal; the procedure durations do not represent individually recorded intervals from chamber removal to euthanasia. Subdermal needle electrodes were positioned as follows: the reference electrode at the mastoid region of the test ear, the recording electrode at the midline of the vertex, and the ground electrode in the contralateral thigh. Electrode impedance was maintained below 5 kΩ. Click and 4 and 8-kHz tone-burst stimuli were presented through the acoustic tube. Frequency-specific ABR measurements were limited to 4 and 8 kHz because these were the tone-stimulus frequencies available on the recording system. ABR thresholds assessed auditory sensitivity, while wave I amplitude and latency at 90 dB SPL provided measures of peripheral auditory response magnitude and timing. Responses were recorded over a 10-ms analysis window at a stimulus rate of 21/s, band-pass filtered at 100–2,000 Hz, and averaged over 1,024 sweeps. Stimulus intensity was decreased in 10-dB steps and then in 5-dB steps near threshold. The ABR threshold was defined as the lowest stimulus level that produced a reproducible waveform in at least two averaged traces. Wave I latency was measured from stimulus onset to the first positive peak, and wave I peak-to-trough amplitude was measured from peak I to the following negative trough (I–Ia). Latency and amplitude were quantified at 90 dB SPL for the 4 and 8-kHz tone-burst responses. All recordings and auditory evaluations were performed by the same investigator.

### 2.5 Cochlear tissue preparation

Cochlear specimens were obtained through two terminal pathways. Samples for ATP and JC-1 assays were collected after terminal blood collection without prior ABR testing and were processed as described in Section 2.9. Histological specimens were obtained immediately after left-ear ABR recording and euthanasia by cervical dislocation under deep isoflurane anesthesia. One ear was randomly selected for histology in each animal; this was not necessarily the ABR-tested ear. Cochleae were dissected and trimmed of surrounding connective tissue under microscopic guidance. For whole-mount preparations, an opening was made at the cochlear apex before overnight fixation in 4% paraformaldehyde at 4 °C. Samples were then decalcified in 10% EDTA for 1.5 h, the otic capsule and tectorial membrane were removed, and the organ of Corti was dissected into apical, middle, and basal turns. For cryosectioning, cochleae were fixed overnight in 4% paraformaldehyde at 4 °C, decalcified in 10% EDTA for 48 h, cryoprotected in 30% sucrose at 4 °C for 30 min, embedded in OCT, and sectioned at 10 μm using a Leica cryostat (Leica Microsystems, Wetzlar, Germany). Cochleae were oriented for serial sectioning toward the mid-modiolar plane. Exact intervals from euthanasia to tissue isolation, lysis, or fixation were not recorded for individual animals.

### 2.6 Immunofluorescence staining and confocal imaging

Cochlear whole mounts were permeabilized and blocked for 1 h at room temperature in PBS containing 5% normal goat serum (ZSGB-BIO, Beijing, China) and 0.3% Triton X-100 (Sigma-Aldrich, St. Louis, MO, United States). Samples were subsequently incubated overnight at 4 °C with rabbit anti-myosin VIIA (1:300; Proteus Biosciences, Cat. No. 25–6,790) and mouse anti-CtBP2 (clone 16, IgG1; 1:300; BD Biosciences, Cat. No. 612044; RRID: AB_399431). After three washes in PBS, samples were incubated for 2 h at room temperature in the dark with goat anti-rabbit IgG (H + L) Alexa Fluor 647 (1:300; Invitrogen, Cat. No. A-21245) and goat anti-mouse IgG1 Cross-Adsorbed Alexa Fluor 568 (1:300; Invitrogen, Cat. No. A-21124). Cryosections were permeabilized and blocked for 2 h at room temperature in PBS containing 5% normal goat serum and 0.3% Triton X-100. Because the primary antibodies used for 4-HNE and oxidized guanine detection were both raised in mouse, the two markers were stained on separate sections. Sections were incubated overnight at 4 °C with rabbit anti-myosin VIIA together with either mouse anti-4-HNE (clone HNEJ-2, IgG1; 1:200; Abcam, Cat. No. ab48506) or mouse anti-DNA/RNA damage antibody (clone 15A3, IgG2b; 1:300; Abcam, Cat. No. ab62623; RRID: AB_940049). Following washing, sections were incubated for 2 h at room temperature in the dark with goat anti-rabbit IgG (H + L) Alexa Fluor 647 (1:300; Invitrogen, Cat. No. A-21245), together with goat anti-mouse IgG1 Cross-Adsorbed Alexa Fluor 568 for 4-HNE staining (1:300; Invitrogen, Cat. No. A-21124) or goat anti-mouse IgG2b Cross-Adsorbed Alexa Fluor 568 for oxidized guanine staining (1:300; Invitrogen, Cat. No. A-21144). Nuclei were counterstained with DAPI before mounting. Images were acquired using a Leica laser-scanning confocal microscope (Leica Microsystems, Wetzlar, Germany). Whole-mount *z*-stacks were acquired at 0.5-μm intervals. For 4-HNE and 8-OHdG-related fluorescence analysis, z-stacks were acquired at 1-μm intervals using an oil-immersion objective and 1.5 × digital zoom. For each marker, laser power, detector gain, offset, pinhole, pixel size, and scan speed were established using nonsaturated control tissue and were then held constant for all groups within the same staining batch.

### 2.7 Hair-cell survival and CtBP2-positive ribbon quantification

OHC survival and CtBP2-positive puncta were quantified in the same whole-mount preparation from one randomly selected cochlea of each of three mice per group. Three non-overlapping fields were sampled from each of the apical, middle, and basal turns. In each field, an OHC was classified as missing when the myosin VIIA-positive cell body was absent from its expected position. Each field contained an expected total of 33 OHCs across the three rows. Field-level counts were summed within each turn to obtain one missing-cell count and one expected-cell total for each mouse and cochlear turn. OHC survival was calculated as (expected OHCs – missing OHCs)/expected OHCs × 100%. Inner hair cells were also inspected for overt loss. CtBP2-positive presynaptic ribbons were quantified in *n* = 3 mice per group. Approximately 10 myosin VIIA-positive inner hair cells (IHCs) were analyzed in each cochlear turn. CtBP2-positive puncta were evaluated using the DAPI, myosin VIIA, and CtBP2 channels, with individual optical sections examined throughout each z-stack. DAPI labeling identified nuclei, while myosin VIIA labeling delineated IHC morphology. Discrete CtBP2-positive puncta in the basolateral IHC region were counted only when they could be distinguished from nuclear labeling. Nuclear signals, nuclear-adjacent signals that could not be clearly separated from nuclear staining, and faint or poorly defined signals that could not be confidently identified as discrete extranuclear puncta were excluded. The same criteria were applied to all experimental groups and cochlear turns from the initial quantification. The puncta count was first calculated for each IHC and then averaged across all analyzed IHCs to generate one animal-level value for each turn. Individual IHCs were treated as within-animal subsamples. Quantification was performed on coded image files by an investigator blinded to group assignment.

### 2.8 Quantification of 4-HNE and 8-OHdG immunofluorescence

Adjacent or serial cochlear cryosections from the same five mice per group were stained separately for 4-HNE and 8-OHdG-related immunoreactivity. Raw confocal z-stack images were analyzed using ImageJ/Fiji by an investigator blinded to group allocation. For each marker and each mouse, three microscopic fields were imaged in each of the apical, middle, and basal cochlear turns. One field from each turn was included in quantitative analysis, yielding three analyzed fields per mouse. The background-corrected mean fluorescence intensities from these three fields were averaged to obtain a single animal-level value for each marker, with equal weighting of the three cochlear turns. Each mouse was treated as an independent biological replicate. Spatially separated, non-overlapping microscopic fields containing an intact organ of Corti were selected. Fields were matched across groups as closely as possible for cochlear level, tissue orientation, and section morphology. Images showing tissue folding, tearing, incomplete sensory epithelium, or pixel saturation were excluded according to predefined technical criteria before group codes were revealed. Each z-stack was converted to a maximum-intensity projection using the same number of optical sections and a comparable total z-depth for all samples within a given marker comparison. A polygonal region of interest was drawn around the myosin VIIA-positive hair-cell compartment, including the inner and outer-hair-cell regions, while excluding adjacent nonsensory tissue and tissue-free background. ROI placement was guided by the myosin VIIA and DAPI channels and was completed before inspection of the 4-HNE or 8-OHdG-related fluorescence channel. Mean fluorescence intensity was measured in the corresponding marker channel. Background fluorescence was measured in a nearby cell-free region within the same image and subtracted from the ROI mean gray value. For graphical presentation, animal-level values were normalized to the mean of the corresponding ambient-pressure control group, which was defined as 1.0. Inferential analyses were performed using the animal-level background-corrected means before normalization. Thus, each data point represented one independent mouse rather than an individual optical section or microscopic field.

### 2.9 ATP content and mitochondrial membrane potential

Whole-cochlear ATP content was measured using an enhanced ATP assay kit (S0027; Beyotime, Shanghai, China). Fresh bilateral cochlear samples were placed in pre-aliquoted ATP lysis buffer immediately after collection and kept on ice. Tissue was homogenized using approximately 150 μL of lysis buffer per 20 mg tissue. Lysates were incubated on ice for 30 min and centrifuged at 12,000 × g for 5 min at 4 °C. ATP-dependent luminescence was measured using an Infinite M200 Pro multimode microplate reader (Tecan, Männedorf, Switzerland), and ATP concentration was calculated from a standard curve. Protein concentration in the same supernatant was measured using a BCA assay (P0010S; Beyotime). ATP content was expressed as nmol/mg protein. Five mice were analyzed per group. The bilateral cochleae from each mouse were pooled to constitute one biological sample (*n* = 5 biological replicates per group). Each lysate was measured once; no technical replicate wells were included. These measurements represent whole-cochlea ATP content and do not provide cell-type-specific AT*p* values or measure ATP synthesis rates.

Mitochondrial membrane potential was assessed in freshly prepared mixed cochlear-cell suspensions using a JC-1 mitochondrial membrane-potential assay kit (C2006; Beyotime, Shanghai, China). Bilateral cochleae from each mouse were rapidly excised and pooled as one biological sample. Under a dissecting microscope, the vestibular portion, including the vestibule and semicircular canals, was removed. The remaining cochlear tissue was transferred to a Petri dish, kept moist with a small volume of phosphate-buffered saline (PBS), and minced with curved scissors until no gross tissue fragments remained. The minced tissue was digested at room temperature for 10 min in 1 mL of 0.25% trypsin–EDTA (Gibco, Thermo Fisher Scientific, United States), with intermittent gentle trituration using a pipette tip. Digestion was terminated by adding 1 mL of DMEM/F-12 medium supplemented with 10% fetal bovine serum (FBS). The resulting suspension was centrifuged at 300 × g for 5 min at 4 °C, and the cell pellet was resuspended in the same complete medium. Cells were then incubated with JC-1 working solution for 20 min at 37 °C, followed by washing according to the manufacturer’s instructions. Samples were acquired using a BD FACSCelesta flow cytometer and analyzed in FlowJo version 10.9.0. Debris was excluded using a forward-scatter-area versus side-scatter-area gate, and singlets were selected using forward-scatter-area versus forward-scatter-height. Aggregate-high/red-dominant and monomer-high/green-dominant populations were defined on compensated FITC-A versus PE-A plots. All available events passing the cell and singlet gates were included in the analysis. A CCCP-treated aliquot was used as a technical positive control to confirm assay responsiveness and gate direction and was not treated as an independent biological group. Three mice were analyzed per group. Bilateral cochleae from each mouse constituted one biological replicate, whereas individual flow-cytometry events were treated as technical observations. No cell-type-specific identification or sorting was performed; JC-1 results therefore represent mitochondrial-polarization profiles in an unsorted mixed cochlear-cell preparation. Bilateral pooling yielded combined measurements from both cochleae; left–right functional equivalence was not assessed.

### 2.10 Statistical analysis

Statistical analyses were performed using IBM SPSS Statistics version 27.0 (IBM Corp., Armonk, NY, USA) and Python. The exact permutation and fixed-sample sensitivity analyses were implemented in Python. The mouse was the experimental unit for all inferential analyses. Descriptive data are presented as mean ± SD, with individual animal-level observations shown in the figures. Pairwise comparisons were two-sided, and *p* < 0.05 was considered statistically significant. Distributional assumptions were evaluated using Q–Q plots and Shapiro–Wilk tests, and homogeneity of variance was assessed using Levene’s test. No observation was excluded solely on the basis of a statistical outlier test; any technical exclusion was applied using pre-specified criteria and reported with the relevant sample size.

RBC, HGB, and HCT were first analyzed jointly using a one-way multivariate analysis of variance (MANOVA), with exposure group as the between-subject factor. Pillai’s trace was used as the primary multivariate statistic. When the overall multivariate effect was significant, each hematological outcome was examined using one-way ANOVA followed by Dunnett’s test to compare each hypoxia group with the control group. When homogeneity of variance was violated, Welch’s ANOVA followed by Games–Howell comparisons was used.

ABR thresholds were analyzed using a two-way mixed-design ANOVA with exposure group as the between-subject factor and stimulus condition (click, 4 kHz, and 8 kHz) as the within-subject factor. Wave I latency and I–Ia amplitude were analyzed in separate two-way mixed-design ANOVAs with exposure group as the between-subject factor and frequency (4 and 8 kHz) as the within-subject factor. For the three-level threshold factor, sphericity was evaluated using Mauchly’s test, and Greenhouse–Geisser-corrected degrees of freedom were reported when sphericity was violated. Prespecified simple-effect analyses compared each hypoxia group with the control group within each stimulus condition or frequency using Dunnett-adjusted comparisons; when variance heterogeneity was substantial, Welch’s ANOVA with Games–Howell comparisons was used for the corresponding simple effect. Because only one ear was tested per mouse, no ear-level repeated or nested factor was included.

OHC loss was calculated for each mouse and cochlear turn by pooling the three sampled fields (99 expected OHC positions per turn). The mouse was the independent unit. Between-group differences in the three-turn loss profile were assessed by an exact Euclidean-distance permutation MANOVA, enumerating12 complete mouse profiles to four groups of three. Complete profiles were permuted together to preserve within-mouse dependence. Exploratory regional tests were adjusted across the three turns using Holm’s method. Individual mouse observations and mean ± SD were reported.

CtBP2-positive puncta were analyzed using a linear mixed model fitted by restricted maximum likelihood, with exposure group, cochlear turn, and their interaction as fixed effects and a compound-symmetry covariance structure for repeated measurements within mouse. To examine group differences within individual turns, separate one-way ANOVAs were fitted for the apical, middle, and basal turns, followed by two-sided Dunnett comparisons of each hypoxia group with the control group. The mouse was the experimental unit, with one animal-level mean per turn. Dunnett adjustment controlled the three control-referenced comparisons within each turn; these regional comparisons were exploratory and did not provide simultaneous error control across all three turns.

Animal-level background-corrected 4-HNE fluorescence intensity and ATP content were analyzed using one-way ANOVA followed by Dunnett comparisons with control. Because of variance heterogeneity, 8-OHdG-related fluorescence intensity was analyzed using Welch’s ANOVA followed by Games–Howell comparisons. JC-1 gate percentages were converted to proportions and logit-transformed before inference; raw percentages were displayed in the figures. The monomer-high and aggregate-high fractions were evaluated separately using Welch’s ANOVA followed by Games–Howell comparisons. The monomer-high fraction was the primary JC-1 outcome, and the aggregate-high fraction was a complementary secondary outcome; the two fractions were not interpreted as independent biological effects.

Exploratory all-pair analyses were added or reported in full for ABR measures, CtBP2-positive puncta, 4-HNE and 8-OHdG-related fluorescence, ATP content, and JC-1 fractions (Supplementary Table S1). All six comparisons among the four groups are reported for each outcome and, where applicable, each stimulus condition, frequency, or cochlear turn. Tukey’s HSD procedure was used for ABR thresholds within each stimulus condition, wave I latency at 4 kHz, amplitude at 8 kHz, 4-HNE, ATP, and CtBP2 separately within each turn. Games–Howell comparisons were used for latency at 8 kHz, amplitude at 4 kHz, and 8-OHdG-related fluorescence, and for logit-transformed JC-1 proportions. Fluorescence analyses used animal-level background-corrected values before control normalization. The ABR mixed-design ANOVAs and the CtBP2 linear mixed model were retained; within-condition or within-turn comparisons used one value per animal. Mean differences, multiplicity-adjusted 95% confidence intervals, and adjusted P values are reported; Games–Howell intervals are approximate. Multiplicity adjustment covers the six comparisons within each stated family, with no additional adjustment across frequencies, turns, or endpoints. The two JC-1 fractions were not regarded as independent biological effects. The original control-referenced comparisons were retained. A Games–Howell sensitivity analysis was also examined for the 4-kHz threshold comparisons. These supplementary analyses describe differences between independent terminal-assessment groups and do not establish longitudinal recovery.

Fixed-sample sensitivity was evaluated for one specified pairwise contrast at 80% marginal power with four groups of three mice. CtBP2 calculations assumed normal observations, equal population variances, and two-sided Dunnett adjustment at a familywise alpha of 0.05 for the three control comparisons within one turn. JC-1 calculations used the four-group Games–Howell decision rule at a nominal alpha of 0.05, assuming normal logit-transformed observations and exposed-to-control population SD ratios of 1 or 2. Standardized differences were defined using the common population SD for CtBP2 and the square root of the mean of the two population variances for JC-1. Calculations used hypothetical population effects, did not impose an additional omnibus-test significance requirement, and did not estimate power for the overall mixed model or interaction.

## 3 Results

The staggered-entry exposure paradigm is summarized in Figure 1. Animals were assigned to nominal 3-, 15-, and 30-day exposure groups, with terminal assessments and tissue collection completed within a period of less than 24 h.

**Figure 1:**
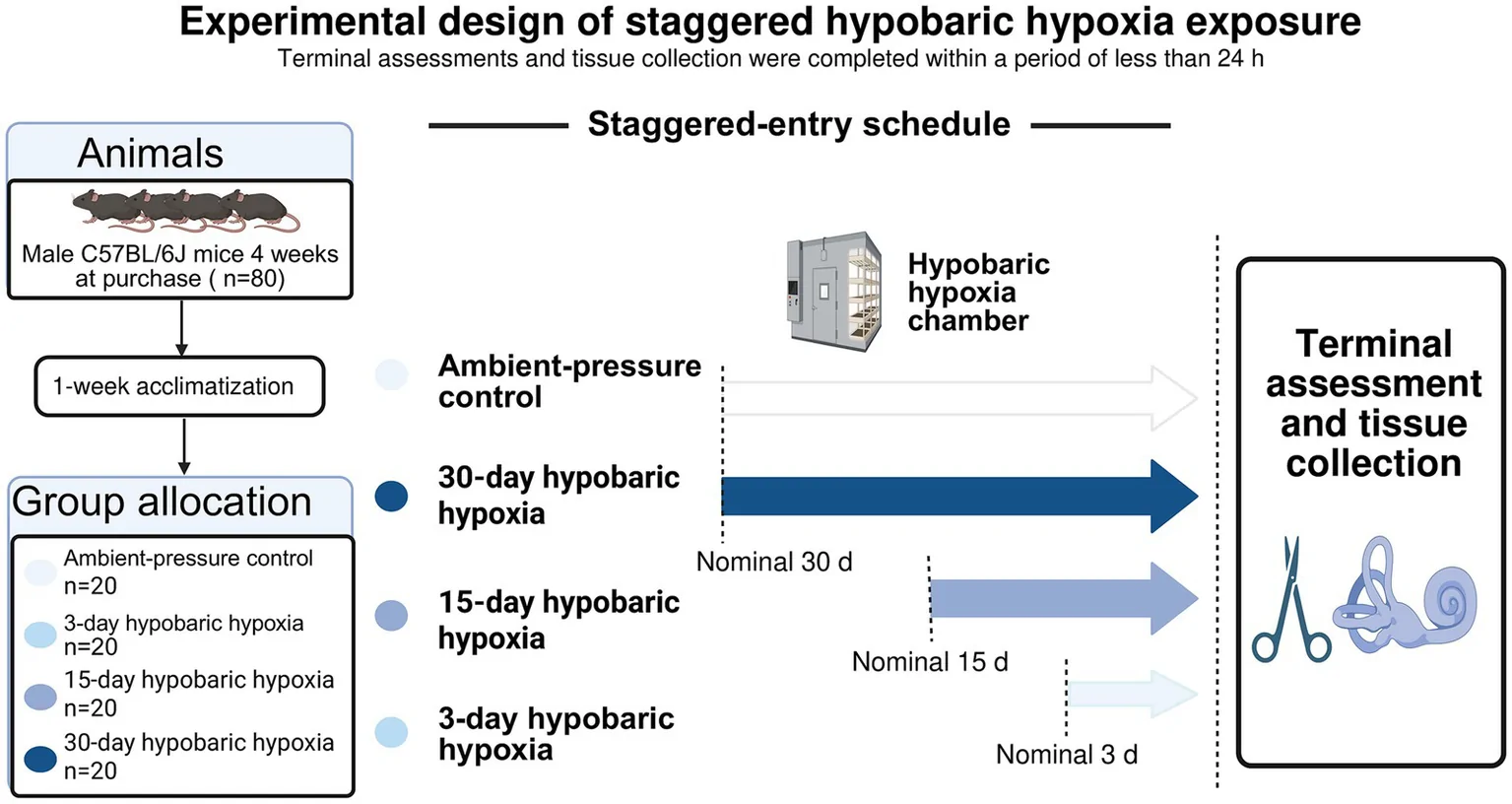
Staggered chamber entry and terminal-assessment schedule. Eighty male C57BL/6J mice were purchased at 4 weeks of age and acclimatized for 1 week before the staggered exposure schedule began, at approximately 5 weeks of age. Mice were assigned to an ambient-pressure control group or nominal 3, 15, and 30-day hypobaric-hypoxia exposure groups (*n* = 20 per group); individual exposure groups entered the chamber at different ages. Chamber entry occurred on 2 June, 21 May, and 6 May 2026, respectively. Terminal assessments and tissue collection were completed within a period of less than 24 h on 5–6 June 2026. Duration labels denote planned exposure groups; actual exposure ended when each animal was removed from the chamber. ABR animals were transferred in pairs while the main chamber remained hypobaric. Control mice remained under local ambient conditions in Xining (approximately 2,260 m), and the simulated exposure altitude was approximately 5,500 m. Created in BioRender. Zhang, Q. (2026) https://BioRender.com/h6iymq1.

### 3.1 Hypobaric hypoxia elicited a systemic hematological response

Exposure group significantly affected the combined hematological profile (Pillai’s trace = 1.389, *F* (9, 108) = 10.35, *p* < 0.001). Follow-up analyses demonstrated group differences in red blood cell count (RBC; *F* (3, 36) = 71.86, *p* < 0.001), hemoglobin concentration (HGB; Welch *F* (3, 18.16) = 545.90, *p* < 0.001), and hematocrit (HCT; *F* (3, 36) = 97.88, *p* < 0.001). Compared with ambient-pressure controls, mice exposed to hypobaric hypoxia for 3, 15, or 30 days had higher RBC, HGB, and HCT values (all adjusted *p* < 0.001; Table 1 and Figure 2). Mean HGB and HCT values were highest at 15 days, whereas the corresponding 30-day values remained above control levels. These findings demonstrate a systemic hematological response to the exposure protocol.

**Table 1:** Hematological indices after hypobaric hypoxia exposure.

Variable	Ambient-pressure control	3d	15d	30d
RBC (×10^12^/L)	9.77 ± 0.22	11.31 ± 0.31^***^	12.73 ± 0.44^***^	12.69 ± 0.87^***^
HGB(g/L)	168.40 ± 2.12	199.00 ± 4.22^***^	236.10 ± 7.62^***^	222.30 ± 4.85^***^
HCT(%)	44.60 ± 0.69	52.69 ± 1.27^***^	62.57 ± 2.33^***^	59.04 ± 4.23^***^

Values are mean ± SD; *n* = 10 mice per group. RBC and HCT were analyzed by one-way ANOVA followed by Dunnett comparisons with the ambient-pressure control. HGB was analyzed by Welch ANOVA followed by Games–Howell comparisons. All three hypoxia groups differed from control at *p* < 0.001 for each variable.

**Figure 2:**
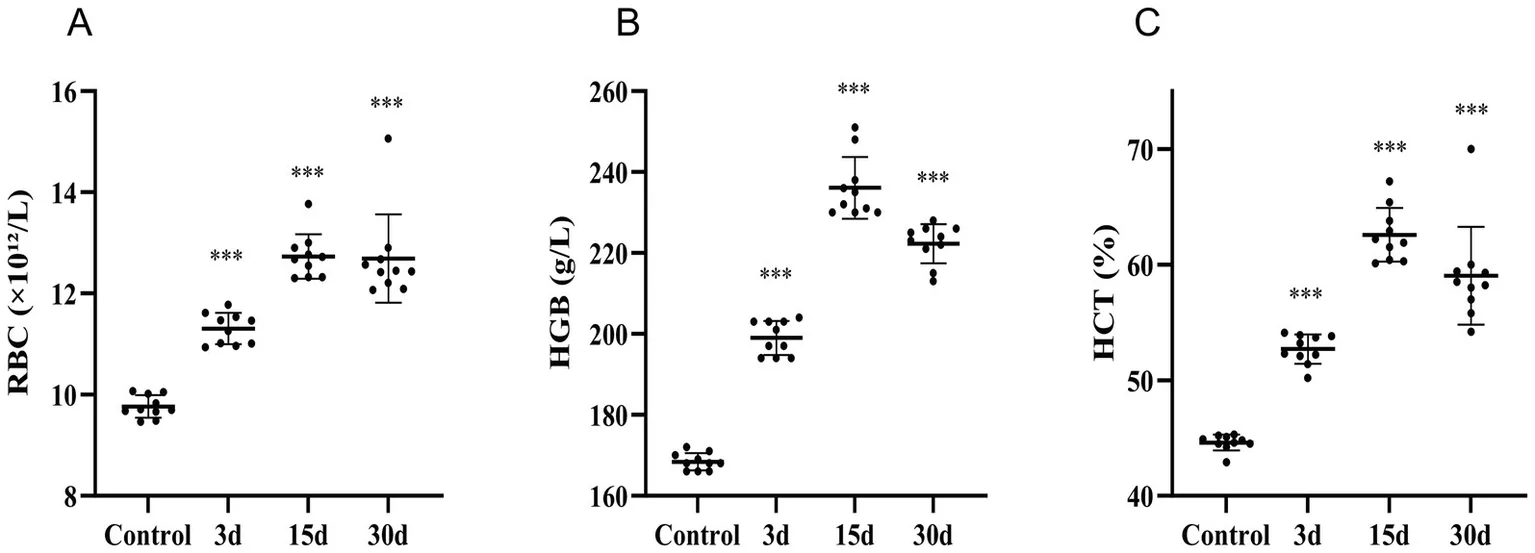
Hypobaric hypoxia increased red blood cell count, hemoglobin concentration, and hematocrit. **(A)** Red blood cell count (RBC). **(B)** Hemoglobin concentration (HGB). **(C)** Hematocrit (HCT). Each symbol represents one mouse; horizontal lines and error bars denote mean ± SD (*n* = 10 mice per group). RBC and HCT were analyzed by one-way ANOVA followed by Dunnett comparisons with the ambient-pressure control; HGB was analyzed by Welch ANOVA followed by Games–Howell comparisons. ***p < 0.001 versus ambient-pressure control.

### 3.2 Exposure-duration-associated changes in ABR thresholds and wave I measures

For ABR thresholds, two-way mixed-design ANOVA revealed significant main effects of exposure group (*F* (3,36) = 33.82, *p* < 0.001) and stimulus condition (*F* (2,72) = 88.36, *p* < 0.001), without a significant Group × Stimulus condition interaction (*F* (6,72) = 1.66, *p* = 0.144). Control-focused comparisons showed that click thresholds were elevated in the 15 and 30-day groups (both *p* < 0.001). At 4 kHz, thresholds were elevated after 3 days (*p* = 0.018), 15 days (*p* < 0.001), and 30 days (*p* < 0.001), whereas at 8 kHz a significant threshold elevation was observed only in the 15-day group (*p* < 0.001) (Figure 3 and Table 2). For wave I latency measured at 90 dB SPL, significant main effects of exposure group (*F* (3,36) = 9.43, *p <* 0.001) and frequency (*F* (1,36) = 36.69, *p* < 0.001) were detected, whereas the Group × Frequency interaction was not significant (*F* (3,36) = 1.91, *p* = 0.145). Wave I latency was prolonged in all three hypoxia groups at both 4 and 8 kHz compared with the ambient-pressure control group (Figure 3B). Analysis of wave I peak-to-trough amplitude revealed significant effects of exposure group (*F* (3,36) = 11.45, *p* < 0.001) and frequency (*F* (1,36) = 167.49, *p* < 0.001), together with a significant Group × Frequency interaction (*F* (3,36) = 6.45, *p* = 0.001). Simple-effect comparisons showed that the 15-day group had a lower I–Ia amplitude than controls at both 4 kHz (*p* = 0.014) and 8 kHz (*p* < 0.001), whereas the 3 and 30-day groups did not differ significantly from controls (Figure 3C). Because the Group × Stimulus interaction for thresholds and the Group × Frequency interaction for latency were not significant, the pattern of within-condition significance should not be interpreted as formal evidence of stimulus or frequency-specific exposure effects.

**Figure 3:**
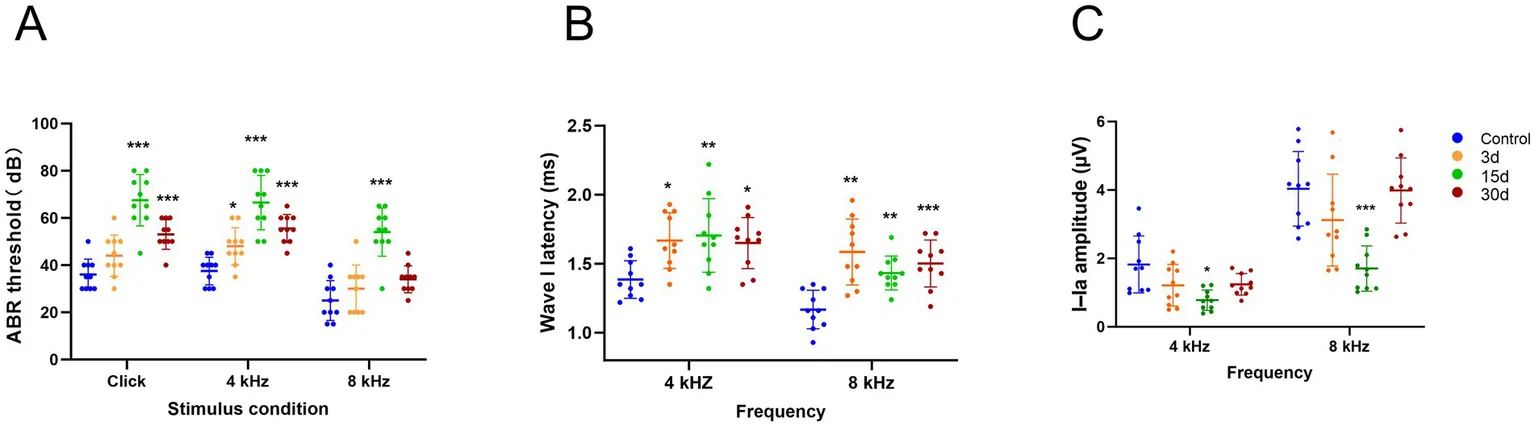
Hypobaric hypoxia altered auditory function assessed by auditory brainstem responses (ABRs). **(A)** ABR thresholds for click, 4-kHz, and 8-kHz stimuli. **(B)** Wave I latency measured at 90 dB SPL for 4 and 8-kHz tone-burst stimuli. **(C)** Wave I peak-to-trough (I–Ia) amplitude measured at 90 dB SPL for 4 and 8-kHz tone-burst stimuli. Each symbol represents one mouse; data are mean ± SD (*n* = 10 mice per group; left ear only). Outcomes were analyzed using two-way mixed-design ANOVA followed by prespecified within-condition comparisons of each hypoxia group with the ambient-pressure control. *p < 0.05, **p < 0.01, and ***p < 0.001 versus ambient-pressure control within the same stimulus condition or frequency. Control comparisons used Dunnett’s procedure except for 8-kHz latency and 4-kHz amplitude, which used Games–Howell comparisons. Complete exploratory all-pair results are provided in Supplementary Table S1; the symbols retain the control-referenced comparisons. Group colors are consistent across panels: control, blue; 3 days, orange; 15 days, green; and 30 days, red.

**Table 2:** Auditory brainstem response measurements after hypobaric hypoxia exposure.

Outcome	Stimulus/frequency	Ambient-pressure control	3 d	15 d	30 d
ABR threshold (dB SPL)	Click	36.0 ± 6.6	44.0 ± 8.8	67.5 ± 10.9^***^	53.0 ± 6.3^***^
ABR threshold (dB SPL)	4 kHz	37.5 ± 5.9	48.0 ± 7.9^*^	66.5 ± 11.6^***^	55.5 ± 6.0^***^
ABR threshold (dB SPL)	8 kHz	25.0 ± 8.5	30.0 ± 10.0	54.0 ± 10.2^***^	34.0 ± 5.7
Wave I latency (ms)	4 kHz, 90 dB SPL	1.386 ± 0.136	1.668 ± 0.201^*^	1.705 ± 0.267^**^	1.650 ± 0.185^*^
Wave I latency (ms)	8 kHz, 90 dB SPL	1.168 ± 0.140	1.585 ± 0.238^**^	1.433 ± 0.122^**^	1.502 ± 0.170^***^
I–Ia amplitude (μV)	4 kHz, 90 dB SPL	1.824 ± 0.833	1.213 ± 0.608	0.780 ± 0.297^*^	1.242 ± 0.315
I–Ia amplitude (μV)	8 kHz, 90 dB SPL	4.038 ± 1.088	3.120 ± 1.340	1.705 ± 0.661^***^	3.985 ± 0.954

Values are mean ± SD; *n* = 10 mice per group, with the left ear analyzed per mouse. Wave I latency and I–Ia amplitude were measured at 90 dB SPL. *p < 0.05, **p < 0.01, ***p < 0.001 versus ambient-pressure control within the same stimulus condition or frequency.

In exploratory Tukey-adjusted comparisons, the 15-day group had higher thresholds than the 3-day group for click, 4-kHz, and 8-kHz stimuli (all *p* < 0.001), and higher thresholds than the 30-day group for click (*p* = 0.0023), 4 kHz (*p* = 0.0233), and 8 kHz (*p* < 0.001). The 4-kHz difference between the 30-day and 15-day groups was −11.0 dB (Tukey-adjusted 95% CI, −20.83 to −1.17), but was sensitive to the variance assumption (Games–Howell sensitivity *p* = 0.0778; 95% CI, −23.02 to 1.02). Wave I amplitude was higher at 30 days than at 15 days at 4 kHz (Games–Howell-adjusted *p* = 0.0163) and 8 kHz (Tukey-adjusted *p* < 0.001); at 8 kHz, amplitude was also lower at 15 days than at 3 days (*p* = 0.0217). No latency comparison between hypoxia groups was significant at either frequency, and none of the seven ABR outcomes differed significantly between the 3-day and 30-day groups. Complete estimates and confidence intervals are provided in Supplementary Table S1.

### 3.3 Hair-cell survival remained largely preserved after hypobaric hypoxia

Representative myosin VIIA-stained whole mounts showed continuous inner-hair-cell rows in all groups and only rare outer-hair-cell (OHC) losses across the apical, middle, and basal turns (Figure 4). OHC survival remained high across all groups and cochlear turns (Figure 5A). The exact mouse-level permutation test did not detect an overall between-group difference in the three-turn loss profile (pseudo-*F* = 1.205; *p* = 0.396). Exploratory regional tests were also nonsignificant after Holm adjustment (all adjusted *p* ≥ 0.604). These findings do not establish equivalence or exclude smaller group differences. IHC integrity was evaluated descriptively rather than by formal survival analysis.

**Figure 4:**
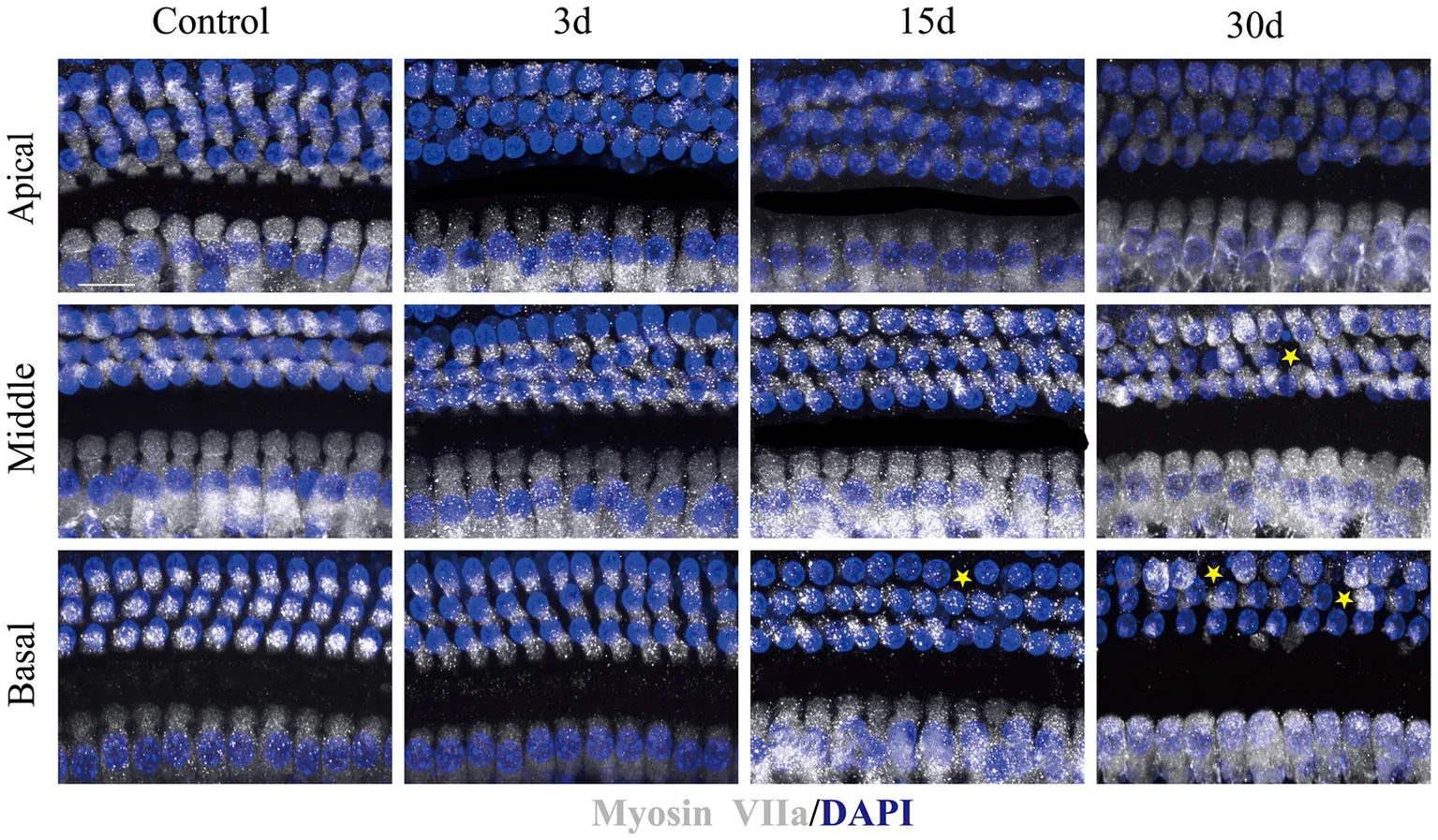
Representative cochlear whole mounts showing preservation of hair-cell architecture after hypobaric hypoxia. Merged images show myosin VIIA detected with Alexa Fluor 647 and displayed in gray, together with DAPI-labeled nuclei in blue. Representative fields from the apical, middle, and basal turns are shown for each group. Yellow asterisks indicate rare missing outer hair cells. Scale bar, 20 μm. The basal-turn control field is derived from the same multichannel confocal z-stack used for the corresponding basal-turn control overview panel in Figure 6. Only the myosin VIIA and DAPI channels are displayed here.

**Figure 5:**
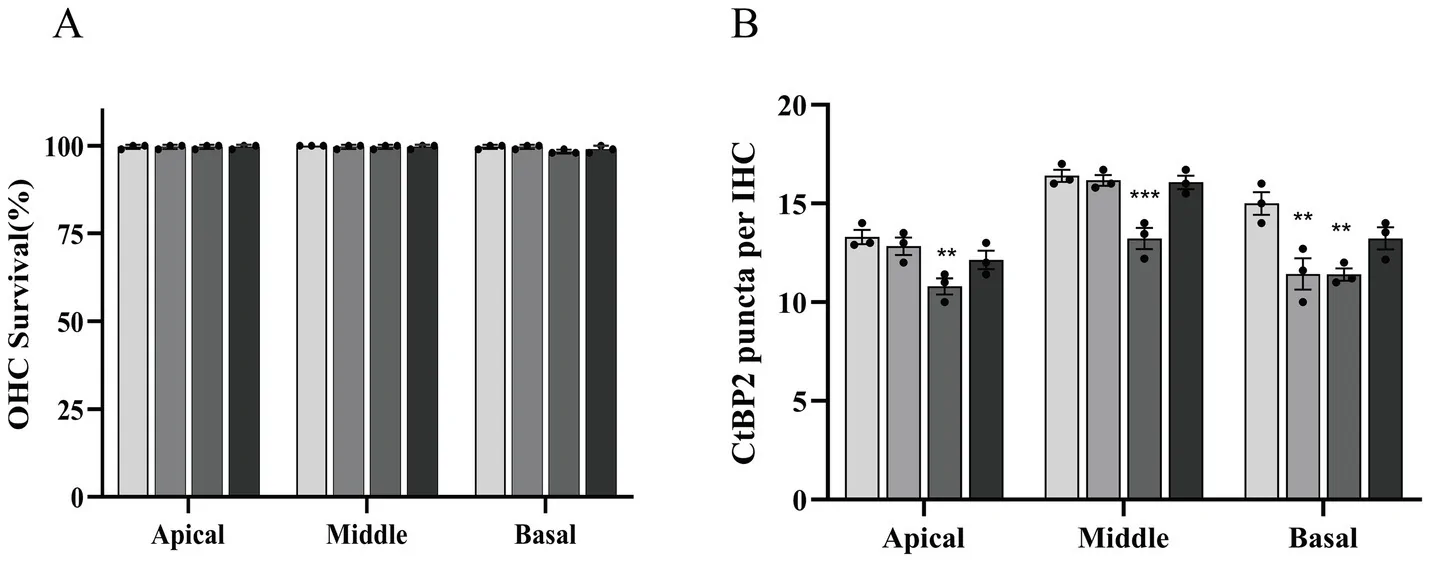
OHC survival and CtBP2-positive puncta across cochlear turns. **(A)** OHC survival in the apical, middle, and basal turns, calculated from three sampled fields containing 99 expected OHC positions per mouse and turn. **(B)** Mean number of CtBP2-positive puncta per IHC in each turn. Each symbol represents one mouse, and the same three mice per group contributed measurements from all three turns and to both endpoints. Data are mean ± SD. OHC group differences were evaluated by an exact permutation analysis of complete three-turn mouse profiles (overall *p* = 0.396). CtBP2-positive puncta were analyzed using a linear mixed model, with regional comparisons obtained from separate one-way ANOVAs followed by two-sided Dunnett tests. Dunnett adjustment applied to the three control-referenced comparisons within each turn. **p < 0.01 and ***p < 0.001 versus control within the same turn. Exploratory all-pair CtBP2 comparisons, adjusted using Tukey’s procedure separately within each turn, are provided in Supplementary Table S1; the symbols in this figure retain the original control-referenced comparisons. Within each turn, bars from left to right represent control, 3 days, 15 days, and 30 days.

### 3.4 CtBP2-positive presynaptic ribbons were reduced in a cochlear-turn-dependent manner

Despite the absence of overt IHC loss or a statistically detectable between-group difference in OHC survival, the number of CtBP2-positive presynaptic ribbon puncta differed across exposure groups and cochlear turns (Figure 6 and Figure 5B). Linear mixed-model analysis showed significant effects of exposure group [*F* (3, 8) = 27.34, *p* < 0.001], cochlear turn [*F* (2, 16) = 49.51, *p* < 0.001], and a significant Group × Turn interaction (*F* (6,16) = 3.07, *p* = 0.034). Compared with the ambient-pressure control group, the 15-day group had fewer CtBP2-positive puncta per IHC in the apical (*p* = 0.0077), middle (*p <* 0.001), and basal (*p =* 0.0060) turns. The 3-day group showed a significant reduction only in the basal turn (*p =* 0.0063), whereas the 30-day group did not differ significantly from controls in any cochlear turn. For the 30-day versus control comparisons, the mean differences in CtBP2-positive puncta per IHC were −1.17 in the apical turn (within-turn Dunnett-adjusted 95% CI, −2.88 to 0.55), −0.33 in the middle turn (−1.87 to 1.21), and −1.77 in the basal turn (−4.13 to 0.60). These intervals remain compatible with reductions in puncta counts despite nonsignificant comparisons. Exploratory Tukey-adjusted comparisons between hypoxia groups showed fewer CtBP2-positive puncta in the 15-day than in the 3-day group in the apical (*p* = 0.0371) and middle (*p* = 0.0025) turns. In the middle turn, the 30-day group had more puncta than the 15-day group (*p* = 0.0031). The other within-turn comparisons between hypoxia groups were not significant (all adjusted *p* ≥ 0.1922). Effect estimates and confidence intervals for all comparisons are provided in Supplementary Table S1; these findings are exploratory and do not establish restoration of ribbon counts within individual animals.

**Figure 6:**
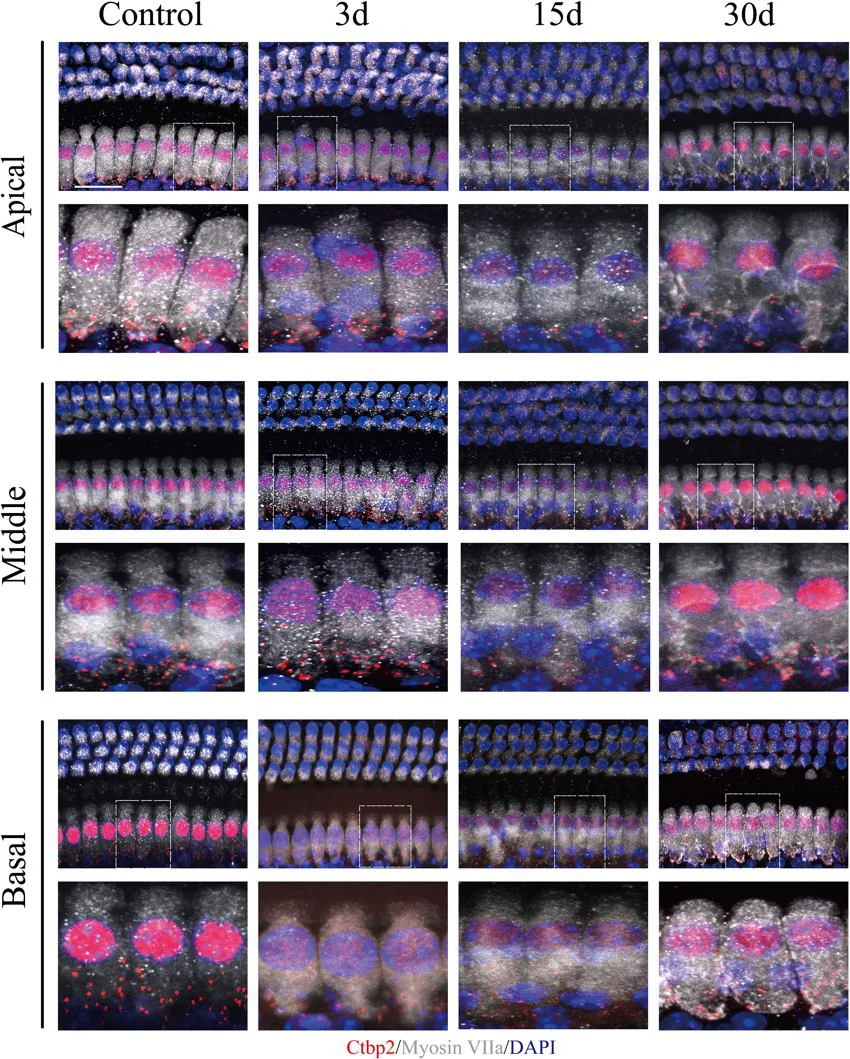
Representative whole-mount immunofluorescence images of CtBP2-positive presynaptic ribbons in inner hair cells. Merged images show CtBP2 detected with Alexa Fluor 568 and displayed in red, myosin VIIA detected with Alexa Fluor 647 and displayed in gray, and DAPI in blue. For each cochlear turn, the upper panels show the organ of Corti and the boxed inner-hair-cell region is shown at higher magnification in the lower panels. Overview scale bar, 20 μm. The basal-turn control overview panel corresponds to the same multichannel confocal z-stack shown in Figure 4, with the CtBP2 channel additionally displayed.

### 3.5 Hypobaric hypoxia increased 4-HNE and 8-OHdG-related immunoreactivity in the organ of Corti

Hypobaric hypoxia increased oxidative-damage-related immunoreactivity in the organ of Corti. Background-corrected 4-HNE fluorescence intensity differed significantly among groups (*F* (3,16) = 34.53, *p* < 0.001). Dunnett-adjusted comparisons showed higher 4-HNE immunoreactivity in the 3-, 15-, and 30-day groups than in the ambient-pressure control group (all *p <* 0.001), with the highest group mean observed after 15 days of exposure (Figure 7A and Figure 7B). Because the homogeneity-of-variance assumption was violated for 8-OHdG-related immunoreactivity, Welch’s ANOVA was used and demonstrated a significant group effect (Welch *F* (3,8.29) = 21.33, *p* < 0.001). Games–Howell comparisons showed increased 8-OHdG-related immunoreactivity in the 3-day (*p* = 0.0057) and 15-day (*p =* 0.0078) groups, whereas the 30-day group did not differ from controls (*p =* 0.982) (Figure 7A and Figure 7C). Exploratory comparisons showed higher 4-HNE immunoreactivity at 15 days than at 3 days (Tukey-adjusted *p* = 0.0006) and 30 days (*p* = 0.0043), whereas the 3 and 30-day groups did not differ significantly (*p* = 0.7580). For 8-OHdG-related immunoreactivity, the 30-day group had lower values than the 3-day (Games–Howell-adjusted *p* = 0.0059) and 15-day groups (*p* = 0.0076); the 3 and 15-day groups did not differ significantly (*p* = 0.5985). Complete estimates and confidence intervals are provided in Supplementary Table S1. Because clone 15A3 recognizes oxidized guanine species associated with both DNA and RNA, the signal does not specifically identify mitochondrial DNA damage.

**Figure 7:**
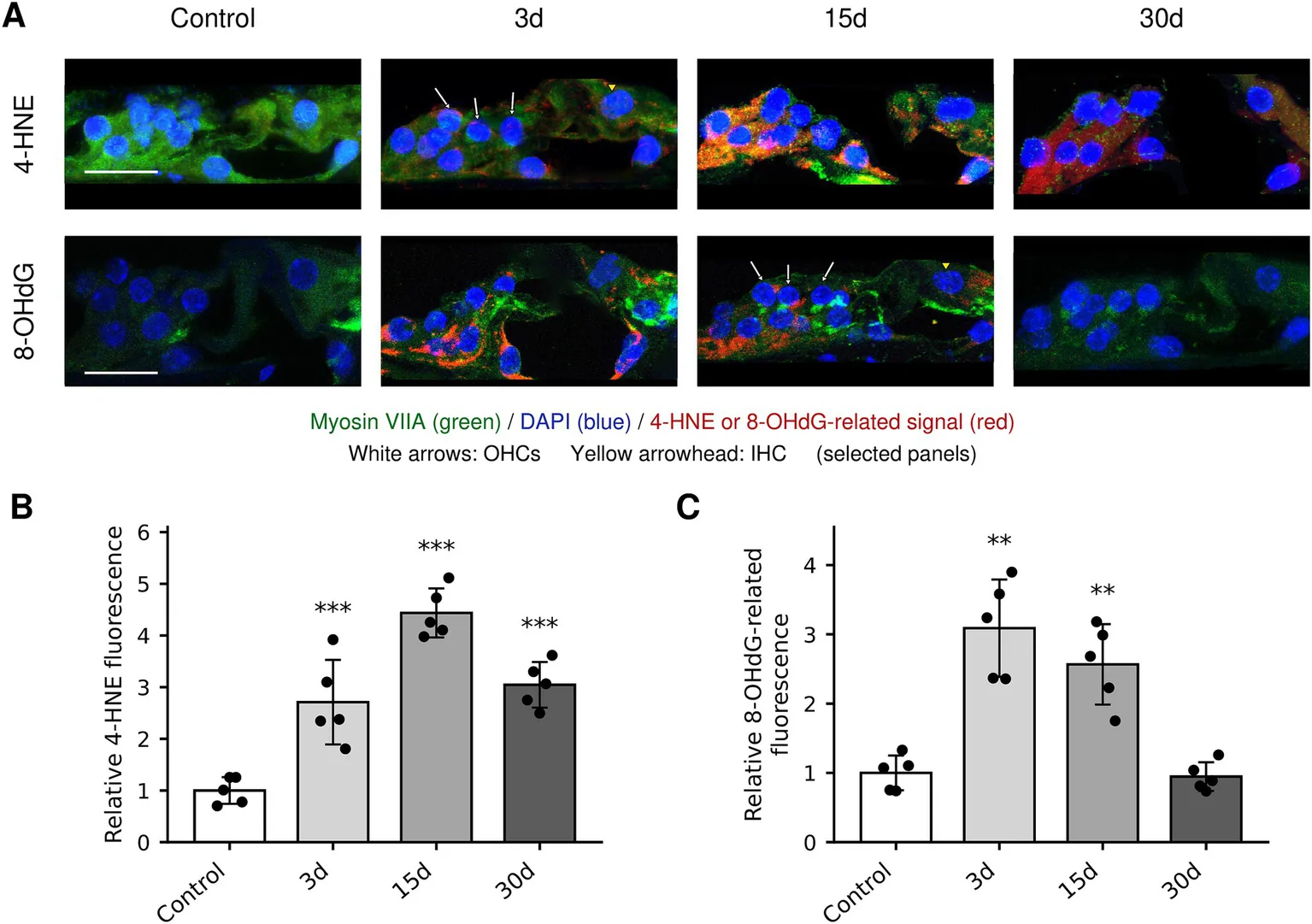
Hypobaric hypoxia increased 4-HNE and 8-OHdG-related immunoreactivity in the organ of Corti. **(A)** Representative confocal images from the middle turn of serial cochlear cryosections oriented toward the mid-modiolar plane. Merged images show myosin VIIA (Alexa Fluor 647, displayed in green), 4-HNE or 8-OHdG-related immunoreactivity (Alexa Fluor 568, red), and DAPI (blue). White arrows indicate outer hair cells, and yellow arrowheads indicate inner hair cells in the annotated panels. Scale bars, 10 μm. **(B,C)** Quantification of 4-HNE **(B)** and 8-OHdG-related **(C)** immunoreactivity. For each marker, one field from each of the apical, middle, and basal turns was analyzed per mouse. The three background-corrected field values were averaged into one animal-level value, then normalized to the mean of the corresponding control group for display. Each point represents one mouse; *n* = 5 mice per group; mean ± SD. 4-HNE was analyzed using one-way ANOVA followed by Dunnett comparisons; 8-OHdG-related immunoreactivity was analyzed using Welch’s ANOVA followed by Games–Howell comparisons. **p < 0.01 and ***p < 0.001 versus ambient-pressure control. Complete exploratory pairwise comparisons are reported in Supplementary Table S1; figure symbols retain the control-referenced comparisons.

### 3.6 Hypobaric hypoxia altered mitochondrial membrane potential and reduced cochlear ATP content

JC-1 flow cytometry revealed a marked shift in mitochondrial-polarization-associated populations after 30 days of hypobaric hypoxia. The monomer-high fraction differed significantly among groups when logit-transformed proportions were analyzed using Welch’s one-way ANOVA [*F* (3, 3.61) = 121.20, *p* < 0.001]. Compared with the ambient-pressure control group, the monomer-high fraction was higher in the 30-day group (32.70 ± 3.03% vs. 3.58 ± 1.84%; Games–Howell-adjusted *p* = 0.022), whereas the 3-day (5.59 ± 1.48%; adjusted *p* = 0.497) and 15-day groups (7.37 ± 0.31%; adjusted *p* = 0.239) did not differ significantly from controls. The aggregate-high fraction showed a complementary pattern [*F*(3, 3.45) = 209.82, *p* < 0.001]. Compared with controls, the aggregate-high fraction was lower in the 30-day group (65.46 ± 2.30% vs. 95.83 ± 2.15%; Games–Howell-adjusted *p = 0.028*), whereas no statistically significant differences were observed in the 3-day (93.60 ± 1.68%; adjusted *p* = 0.520) or 15-day groups (91.60 ± 0.17%; adjusted *p* = 0.254) (Figures 8A–C). Because the aggregate-high and monomer-high fractions were largely complementary gated fractions derived from the same JC-1 assay, they were interpreted as two manifestations of the same 30-day shift toward reduced JC-1 aggregation, consistent with mitochondrial depolarization, rather than as independent biological effects. Whole-cochlear ATP content differed markedly among exposure groups (*F* (3,16) = 206.98, *p* < 0.001). Compared with the ambient-pressure control group, ATP content was reduced after 3, 15, and 30 days of hypobaric hypoxia (all Dunnett-adjusted *p* < 0.001). The lowest group mean was observed after 15 days of exposure (Figure 8D).

**Figure 8:**
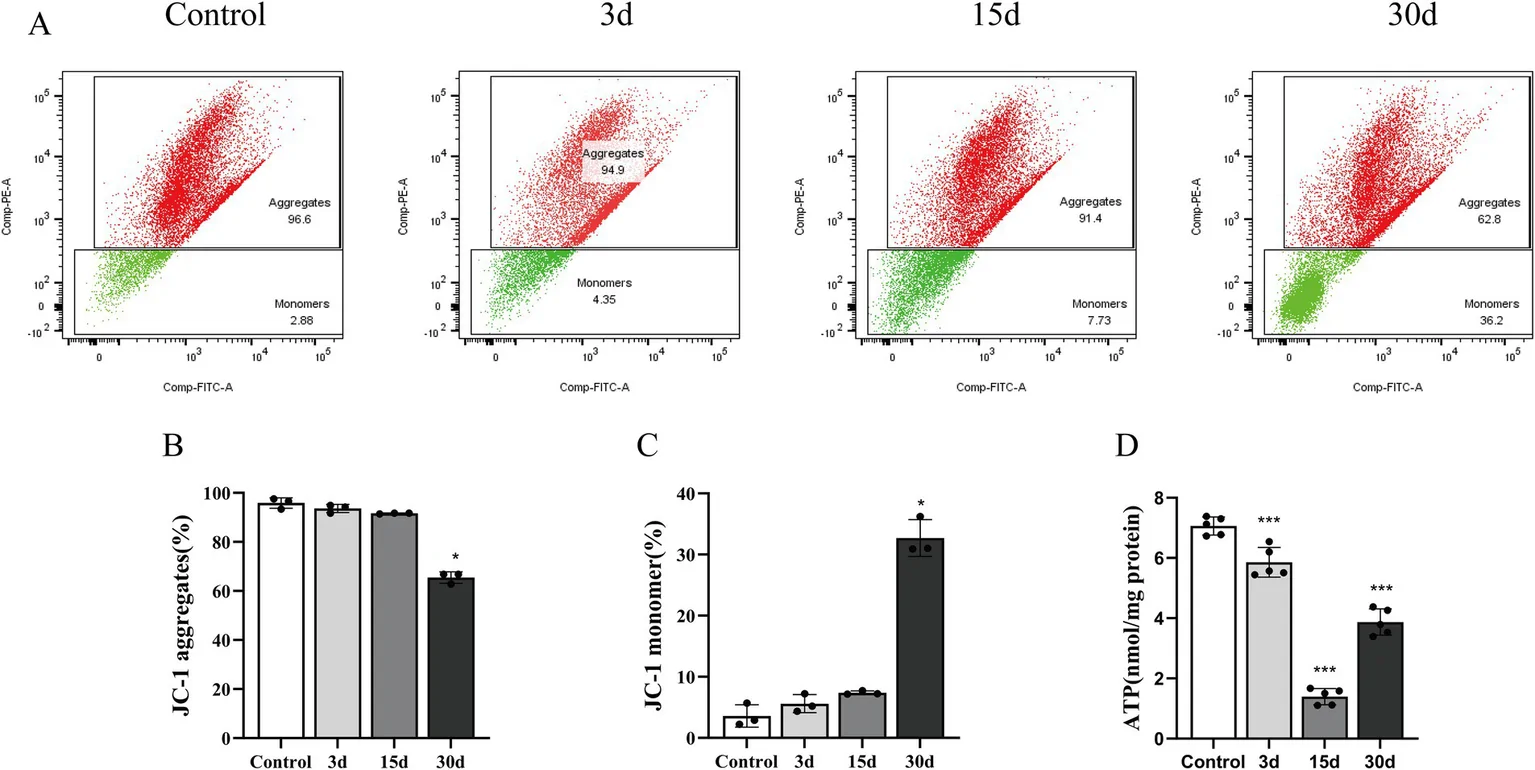
Hypobaric hypoxia altered JC-1-defined mitochondrial polarization and reduced whole-cochlear ATP content. **(A)** Representative JC-1 flow-cytometry plots showing aggregate-high/red-dominant and monomer-high/green-dominant populations in mixed cochlear-cell suspensions. **(B)** Percentage of singlet events within the aggregate-high gate. **(C)** Percentage of singlet events within the monomer-high/depolarized gate. **(D)** Whole-cochlear ATP content normalized to total protein concentration. Bilateral cochleae from each mouse were pooled as one biological sample. Each point represents one mouse; *n* = 3 mice per group for JC-1 analysis and *n* = 5 mice per group for ATP measurement. Data are presented as mean ± SD. Raw JC-1 percentages are shown, whereas inferential analyses were performed on logit-transformed proportions using Welch’s one-way ANOVA followed by Games–Howell comparisons. ATP content was analyzed using one-way ANOVA followed by Dunnett’s test. *p < 0.05 and ***p < 0.001 versus the ambient-pressure control group. Complete exploratory pairwise comparisons for ATP and JC-1 are reported in Supplementary Table S1; the symbols in this figure retain the original control-referenced comparisons.

Exploratory comparisons between hypoxia groups showed a higher JC-1 monomer-high fraction in the 30-day group than in the 3-day and 15-day groups (Games–Howell-adjusted *p* = 0.0041 and *p* = 0.0021, respectively, on the logit scale), with complementary reductions in the aggregate-high fraction (*p* = 0.0068 and *p* = 0.0018). Neither fraction differed significantly between the 3-day and 15-day groups. ATP content was lower in the 15-day group than in the 3-day and 30-day groups, and was lower in the 30-day than in the 3-day group (all Tukey-adjusted *p* < 0.001). The 30-day minus 15-day ATP difference was 2.475 nmol/mg protein (Tukey-adjusted 95% CI, 1.778 to 3.173). These are differences between independent terminal-assessment groups; they do not demonstrate within-animal recovery. The complete pairwise results are provided in Supplementary Table S1.

## 4 Discussion

The present study examined cochlear responses after 3, 15, and 30 days of simulated high-altitude hypobaric hypoxia. Three principal findings emerged. First, hypobaric hypoxia was associated with ABR threshold elevation and prolonged wave I latency, whereas wave I amplitude measured at 90 dB SPL was reduced only in the 15-day group. Second, these physiological abnormalities occurred without overt IHC loss or a statistically detectable between-group difference in OHC survival, whereas CtBP2-positive presynaptic ribbons were reduced in the basal turn after 3 days and across all three cochlear turns after 15 days. Third, the auditory and presynaptic findings were accompanied by increased oxidative-damage-related immunoreactivity, reduced whole-cochlear ATP content, and altered JC-1-defined mitochondrial polarization. Collectively, these findings show auditory and presynaptic abnormalities alongside cochlear oxidative and metabolic changes, despite largely preserved hair-cell bodies.

The hematological findings confirm that the exposure paradigm imposed a biologically effective systemic hypoxic stimulus. RBC count, HGB concentration, and HCT were elevated at all three exposure durations, indicating increased blood oxygen-carrying capacity or hemoconcentration, or a combination of both processes. The early changes observed after 3 days should not be interpreted solely as newly generated erythrocyte mass, because hypoxic exposure can rapidly alter plasma volume before sustained erythropoiesis becomes fully established. With more prolonged exposure, increased erythropoietic activity and expansion of hemoglobin mass and red-cell volume may contribute more substantially to the hematological phenotype (14, 15). The highest group means for HGB and HCT were observed at 15 days, whereas the corresponding 30-day means were lower but remained markedly above control values. Because direct temporal contrasts were not used to establish a statistically significant decrease from 15 to 30 days, this pattern is best described as a non-monotonic hematological response rather than definitive evidence of chronic acclimatization. Nevertheless, the persistent elevation of all three indices confirms that cochlear findings were obtained against a background of sustained systemic hypoxic exposure.

The ABR findings are broadly consistent with previous evidence that reduced oxygen availability affects both auditory sensitivity and neural timing. Children living at approximately 2,800–3,000 m have shown prolonged brainstem-response latencies and reduced wave I and V amplitudes, while prolonged human exposure to higher altitude has also been associated with delayed auditory brainstem responses (16). More recently, simulated exposure of C57BL/6J mice to approximately 6,000 m produced time-dependent auditory abnormalities at the tested low frequencies and dynamic changes in peripheral and central auditory electrophysiology (5). In the present study, mixed-design analyses showed significant main effects of exposure group on ABR threshold and wave I latency, but the Group × Stimulus condition interaction for thresholds and the Group × Frequency interaction for latency were not significant. Accordingly, these outcomes support exposure-associated abnormalities across the tested conditions rather than statistically established stimulus or frequency-specific effects. Wave I amplitude, in contrast, showed a significant Group × Frequency interaction and was lower than control at both 4 and 8 kHz only in the 15-day group. This non-parallel pattern suggests that auditory sensitivity, response timing, and aggregate neural output may show different exposure-duration profiles. However, because wave I amplitude was measured at a fixed level of 90 dB SPL, the amplitude reduction cannot be attributed directly to synaptic loss.

These ABR findings nevertheless require cautious interpretation. Wave I amplitude and latency were measured at a fixed physical level of 90 dB SPL, whereas ABR thresholds differed substantially among groups. Consequently, the effective sensation level was lower in animals with elevated thresholds, and this difference may have contributed to reduced wave I amplitude or prolonged latency. Threshold elevation also complicates the use of wave I amplitude as an isolated estimate of cochlear synapse number (11). Thus, the reduced wave I amplitude in the 15-day group is consistent with impaired auditory-nerve recruitment or synchrony but cannot, by itself, be considered proof of synaptic loss. Future studies should construct wave I amplitude–level and latency–level functions across multiple sound levels and compare responses at both matched SPL and matched sensation levels. Incorporating DPOAEs would additionally help separate changes in cochlear amplification from neural abnormalities.

Potential vulnerability of the cochlear apex to hypoxia warrants consideration when interpreting the ABR findings. An early experimental study in chinchillas described a tendency toward greater hypoxia-associated morphological changes in the apical than basal turn (17). In mice, acute systemic hypoxia produced spatially heterogeneous blood-flow responses within the apical turn (18). These observations suggest that apical regions may be susceptible under some hypoxic conditions, but differences in species, exposure conditions, and endpoints limit extrapolation to the present model. The present data do not establish preferential low-frequency impairment or selectively localize dysfunction to the cochlear apex.

An important observation was that substantial physiological abnormalities occurred without overt IHC loss or a statistically detectable between-group difference in OHC survival. This pattern suggests that functional abnormalities can be present while hair-cell bodies remain largely preserved. Previous high-altitude studies in rats suggested that spiral-ganglion abnormalities can precede extensive hair-cell degeneration, whereas obvious hair-cell loss becomes more prominent after substantially longer residence at altitude (3). Moreover, preservation of myosin VIIA-positive hair-cell bodies does not establish normal OHC electromotility, stereociliary function, ionic homeostasis, or endocochlear potential. Systemic hypoxia can produce spatially heterogeneous alterations in cochlear blood flow, and disruption of cochlear pericytes or the blood–labyrinth barrier can impair blood supply and hearing without requiring immediate sensory-cell death (18, 19). The stria vascularis and cochlear microcirculation therefore remain plausible contributors to the observed threshold elevations. Because the present study did not measure DPOAEs, cochlear microphonic responses, endocochlear potential, or strial morphology, the relative contributions of OHC dysfunction, strial impairment, and neural injury cannot yet be resolved. In addition, although baseline external and middle-ear abnormalities were excluded, post-exposure tympanometry was not performed; therefore, a small pressure-related conductive component cannot be entirely excluded (2).

The CtBP2 results provide the most direct structural evidence of an early presynaptic cochlear abnormality. Synaptic ribbons are required for rapid, sustained, and temporally precise neurotransmitter release from IHCs, and disruption of ribbon organization impairs synchronous auditory signaling (8). In acquired hearing-loss models, degeneration of IHC–auditory-nerve connections can occur before permanent threshold elevation or sensory-cell loss becomes evident (9, 10). In the present study, a significant reduction in CtBP2-positive puncta was detected in the basal turn after 3 days and in all three turns after 15 days, relative to control. A similar dissociation between preserved hair-cell counts and early synaptic or neural injury has been described after synaptopathic noise exposure, during cochlear aging, and after low-dose gentamicin exposure (9, 20, 21). Although these models do not establish an identical mechanism for hypobaric hypoxia, they identify the IHC–auditory-nerve junction as a stress-sensitive cochlear compartment. The co-occurrence of widespread CtBP2 reduction and lower wave I amplitude at 90 dB SPL in the 15-day group is biologically compatible with presynaptic involvement. However, this co-occurrence does not demonstrate that loss of presynaptic ribbons caused the amplitude reduction, because wave I was measured at a fixed SPL and CtBP2 does not identify paired functional synapses. These findings support alterations in presynaptic ribbon-associated labeling but do not establish the number of intact paired synapses or confirm loss of functional IHC–auditory-nerve connections. Future studies should combine CtBP2/GluA2-paired puncta measurements with complementary functional assessments and, where feasible, auditory-nerve terminal or neurofilament labeling. Nuclear and ambiguous CtBP2 signals were excluded using the same criteria across groups. Nevertheless, labeling intensity, antigen detectability, and subcellular distribution could influence the number of confidently identifiable extranuclear puncta. Excluding weak or poorly defined signals may therefore affect quantitative estimates. Nuclear CtBP2 abundance and protein redistribution were not measured, so the present data do not establish stress-induced nuclear translocation or fully distinguish altered puncta detectability from structural ribbon changes.

The early structural and functional findings were not spatially concordant. At 3 days, a significant reduction in CtBP2-positive puncta was detected only in the basal turn, whereas wave I latency was prolonged at both 4 and 8 kHz. The basal-turn finding therefore does not, by itself, explain the latency changes at these low frequencies. With only three histological animals per group, nonsignificant findings in the other turns do not establish that their ribbon counts were unaffected. Changes in ribbon organization or synaptic transmission, auditory-nerve conduction or synchrony, cochlear amplification, or ionic homeostasis could contribute without detectable puncta loss, but these mechanisms were not directly measured. Differences in effective sensation level at the fixed 90-dB-SPL test level may also have contributed to the latency differences. Thus, the present data do not establish a direct spatial or causal correspondence between puncta reduction and early latency prolongation. Histological sampling covered broad cochlear turns rather than individual frequency positions, and equipment limitations restricted tonal ABR testing to 4 and 8 kHz, precluding assessment of higher-frequency dysfunction. Studies in larger cohorts combining broader-frequency ABR recordings, latency–level functions at matched SPL and sensation levels, and frequency-matched histology are needed to clarify the relationship between regional structural and functional abnormalities.

The redox and bioenergetic findings provide a plausible biological context for the auditory and ribbon abnormalities. Hypoxia does not simply suppress mitochondrial activity; changes in electron transport, particularly at mitochondrial complex III, can increase redox signaling and cytosolic ROS generation during cellular oxygen sensing (12). When redox signaling exceeds antioxidant and repair capacity, lipid, protein, and nucleic-acid oxidation may accumulate. In the present study, 4-HNE immunoreactivity was elevated at all three exposure durations, indicating persistent lipid-peroxidation-associated stress, whereas 8-OHdG-related oxidized-guanine immunoreactivity was increased at 3 and 15 days but not at 30 days. Whole-cochlear ATP content was reduced throughout exposure, with the lowest group mean at 15 days, while a marked shift from aggregate-high to monomer-high JC-1 populations was detected at 30 days. Because the JC-1 analysis included only three biological replicates per group, the 30-day mitochondrial-polarization finding should be regarded as exploratory and requires independent replication in a larger cohort. These endpoints do not need to change synchronously: 4-HNE adduct formation, oxidized-guanine accumulation and repair, tissue ATP availability, and mitochondrial membrane polarization represent different biochemical processes and were measured in different cellular preparations. The JC-1 findings do not establish a temporal sequence linking the earlier oxidized-guanine signal to subsequent mitochondrial dysfunction.

The metabolic findings have limited cellular specificity. Whole-cochlear ATP content and JC-1 measurements in unsorted cochlear-cell suspensions cannot identify the cell populations responsible for the observed differences or establish metabolic dysfunction specifically in IHCs, OHCs, or IHC–auditory-nerve synapses. Cell composition may also influence measurements from these mixed preparations, but cell-type contributions were not quantified. Moreover, ATP and JC-1 assays were performed in different animals from those used for ABR and histology. Their coexistence across exposure groups therefore does not establish an individual-level association or a causal link between metabolic and presynaptic or auditory abnormalities. Future studies should combine cell-type-resolved ATP or mitochondrial-polarization measurements with matched functional and structural assessments. Bilateral pooling can obscure asymmetric responses: the pooled values cannot be assigned to either ear, and left–right comparability was not tested.

Experimental studies in other acquired hearing-loss models strengthen the biological plausibility of this relationship. Constitutive ROS overproduction can reduce CtBP2 and other ribbon-associated proteins before extensive hair-cell degeneration (22). In noise-exposed animals, mitochondrial-targeted antioxidant treatment reduced 4-HNE and 8-OHdG signals, improved ATP production, and attenuated ribbon-synapse and auditory-nerve injury (23). The present findings are consistent with a working model in which redox and energetic stress may contribute to presynaptic and neural dysfunction during hypobaric hypoxia. They do not demonstrate that oxidative stress mediated the observed auditory abnormalities or that a specific ROS–mitochondrial pathway was causally responsible for the presynaptic changes. The 4-HNE and 8-OHdG-related measurements were semiquantitative immunofluorescence endpoints; Clone 15A3 recognizes oxidized guanine species associated with DNA and RNA. Staining in our sections was predominantly cytoplasmic; however, this localization alone does not distinguish oxidized DNA from oxidized RNA or establish a mitochondrial origin. ATP was measured in whole-cochlear lysates, and JC-1 was measured in mixed cochlear-cell suspensions rather than isolated IHCs or OHCs. Furthermore, no antioxidant, mitochondrial-protective, or genetic intervention was used. Accordingly, the appropriate conclusion is that auditory and ribbon abnormalities were associated with oxidative and bioenergetic disturbance, not that a specific ROS–mitochondrial pathway has been causally established.

The 30-day findings do not establish cochlear recovery. Exploratory comparisons showed higher ATP content and more middle-turn CtBP2-positive puncta at 30 days than at 15 days, but the apical and basal-turn CtBP2 comparisons were not significant. These differences between independently sampled groups do not demonstrate restoration within individual animals. Furthermore, ATP remained below control levels, 4-HNE and wave I latency remained elevated, and JC-1 polarization remained abnormal at 30 days. Nonsignificant CtBP2 and 8-OHdG comparisons with control do not establish equivalence. The 30-day group also had lower click and 8-kHz thresholds, higher wave I amplitudes at both tested frequencies, and lower 4-HNE and 8-OHdG-related immunoreactivity than the 15-day group. However, the 4-kHz threshold comparison was sensitive to the variance assumption, and wave I latency did not differ significantly between hypoxia groups. These endpoint-specific differences do not establish a temporal peak, an equivalent plateau, or recovery within individual animals. Longer high-altitude rat studies likewise indicate that 30 days may still represent an injury phase, whereas clearer auditory acclimatization or tissue repair becomes evident at approximately 60 days (4, 24). The present findings do not establish a fully acclimatized cochlear state at 30 days. Studies incorporating additional exposure durations and longitudinal auditory measurements could help distinguish persistent injury, functional improvement, and acclimatization.

Several design features strengthen the present study. Staggered chamber entry brought the exposure groups into a narrow terminal-assessment window, limiting age differences at assessment. Auditory physiology, hair-cell survival, presynaptic-ribbon morphology, oxidative-damage-associated immunoreactivity, ATP content, and mitochondrial polarization were also evaluated within a single experimental framework. Nevertheless, the ambient-pressure controls were maintained in Xining at approximately 2,260 m rather than at sea level. The study therefore estimates the incremental effect of simulated exposure to approximately 5,500 m relative to a moderate-altitude background, which is relevant to further ascent from Xining but may underestimate differences relative to sea-level animals. Because the ambient-pressure controls were housed outside the hypobaric chamber and no chamber-matched control was included, the groups differed in both exposure pressure and chamber environment. Confinement, humidity, ventilation, carbon dioxide exposure, and possible pump-generated noise may have contributed to the observed differences; equipment noise is particularly relevant to auditory outcomes. Recording chamber conditions does not exclude these influences. Accordingly, the findings describe outcomes associated with the hypobaric-chamber exposure protocol, and effects specific to hypobaric hypoxia cannot be fully isolated. A chamber-matched ambient-pressure control would improve causal attribution in future studies. The use of young, male C57BL/6J mice limits generalizability. The C57BL/6J background also carries the susceptible *Cdh23^ahl^* allele, which may interact with environmental stressors (25).

Several methodological limitations should be considered. First, ABR measurements were obtained after transfer to local ambient conditions under isoflurane anesthesia with oxygen administration, whereas hematological indices came from a separate subgroup without prior ABR testing. The blood results therefore cannot establish the physiological state of the ABR animals. Staged transfer limited time outside the chamber but did not eliminate reoxygenation: the second mouse in each pair waited awake while the first was tested. Groups were tested sequentially within the terminal-assessment period, so testing time and group assignment were not fully separated. Comparative mouse studies have reported anesthetic-regimen-dependent differences in auditory sensitivity and wave I amplitude (26, 27). These studies do not isolate the contribution of oxygen as a carrier gas in our exposure model. The ABR measurements in the hypoxia groups therefore reflect auditory function during acute reoxygenation and anesthesia, and the contributions of these conditions to the observed group differences cannot be separated. Because histology was performed on a randomly selected ear whereas ABR was recorded from the left ear, functional and histological measurements were not consistently obtained from the same cochlea. This limits direct assessment of structure–function correspondence within individual animals; the present findings therefore support group-level co-occurrence of auditory and histological abnormalities. Second, ABR was measured only at the terminal assessment. Consequently, individual threshold shifts or within-animal changes in wave I measures could not be calculated, and baseline hearing could not be included as a covariate. Otoscopic screening and random allocation do not establish equivalent baseline cochlear or auditory-nerve function. Without baseline recordings, the contribution of pre-existing interindividual variability, particularly in wave I amplitude, to the terminal group differences cannot be quantified. Pre-exposure ABR recordings would strengthen future studies. Fixed-SPL wave I measurements and the absence of amplitude–level functions, DPOAEs, and endocochlear-potential measurements also limit physiological localization. Third, small animal numbers limit precision and sensitivity, particularly for OHC, CtBP2, and JC-1 endpoints. Under the stated sensitivity-analysis assumptions, 80% marginal power for a specified comparison required a standardized difference of approximately 3.12 for within-turn CtBP2 Dunnett comparisons and 5.13 or 5.74 for JC-1 Games–Howell comparisons with population SD ratios of 1 or 2, respectively. These hypothetical calculations illustrate limited sensitivity to smaller effects and do not establish adequate power. OHC survival was also close to the measurement ceiling. Nonsignificant differences, including the 30-day CtBP2 and 8-OHdG comparisons, cannot establish recovery or equivalence to control. Individual post-mortem processing intervals were not recorded, so uniform handling times cannot be verified and effects of tissue-handling time on the measured endpoints cannot be excluded. Finally, the study was observational at the mechanistic level. CtBP2 labeling did not quantify paired synapses, and ATP and JC-1 measurements lacked cell-type specificity. Future studies combining antioxidant or mitochondrial-targeted interventions with paired synapse measurements, wave I amplitude–level functions, DPOAEs, and cell-type-specific metabolic assays could clarify the pathways underlying the observed associations.

## 5 Conclusion

To sum up, simulated high-altitude hypobaric hypoxia was associated with auditory and cochlear abnormalities whose pattern varied across exposure groups and endpoints. ABR thresholds and wave I latency were altered, and wave I amplitude at 90 dB SPL was reduced in the 15-day group, while no overt IHC loss or significant between-group difference in OHC survival was detected. CtBP2-positive presynaptic ribbons were reduced in the basal turn after 3 days and across all cochlear turns after 15 days, and these changes occurred alongside oxidative-damage-related immunoreactivity and reduced cochlear ATP content. Because wave I was assessed at a fixed SPL and CtBP2 marks presynaptic ribbons rather than paired functional synapses, the physiological and structural findings should be interpreted as associated rather than causally linked. Exploratory comparisons identified differences between the 15-day and 30-day groups in several auditory, structural, and biochemical outcomes, but these independent-group findings do not establish within-animal recovery. The metabolic assays also do not localize dysfunction to IHCs or their synapses. These findings support cochlear presynaptic and metabolic abnormalities as early features of hypobaric-hypoxia exposure and provide a rationale for future studies using matched-sensation-level electrophysiology, paired synapse analysis, and mechanistic intervention.

## Data Availability

The raw data supporting the conclusions of this article will be made available by the authors, without undue reservation.
