# Supplementary material for: Exposure-duration-associated auditory dysfunction and cochlear presynaptic ribbon alterations during simulated high-altitude hypobaric hypoxia in mice
Source: Front Neurol. 2026 Oct 15;17:1963563. doi: 10.3389/fneur.2026.1963563 (PMC13716653; doi:10.3389/fneur.2026.1963563)
Supplement: Supplementary file 1 [file Image_1.pdf]

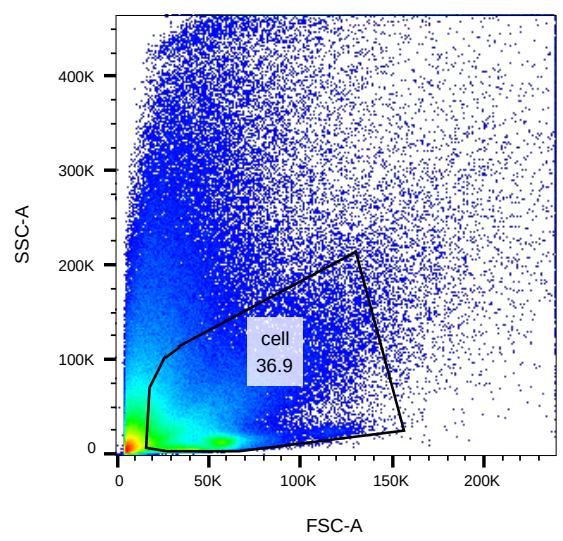

20251028_cccp_003.fcs
Ungated
5.07E5

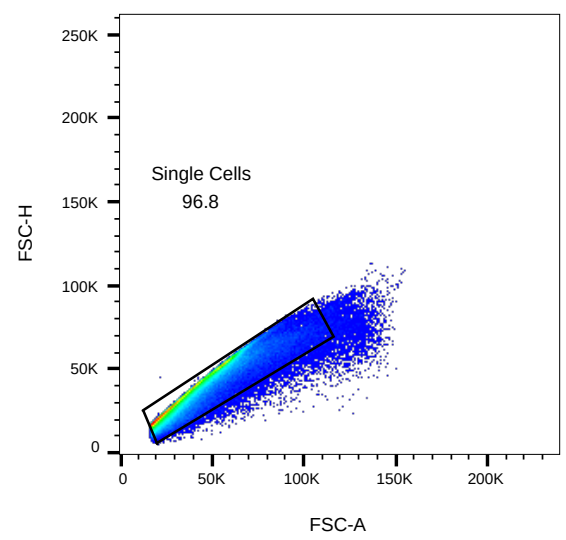

20251028_cccp_003.fcs
cell
186905

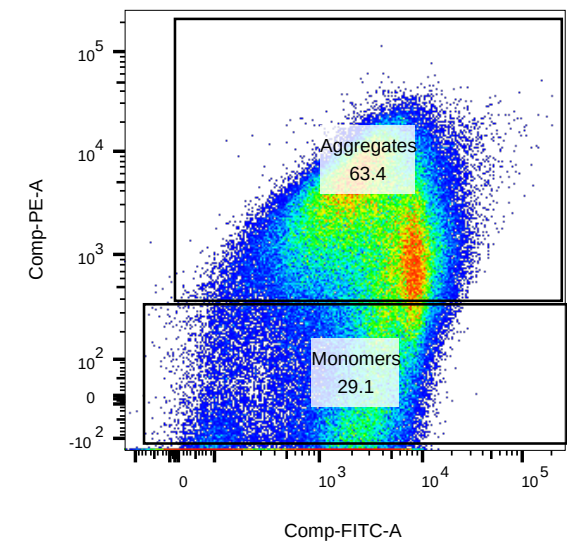

20251028_cccp_003.fcs
Single Cells
180913

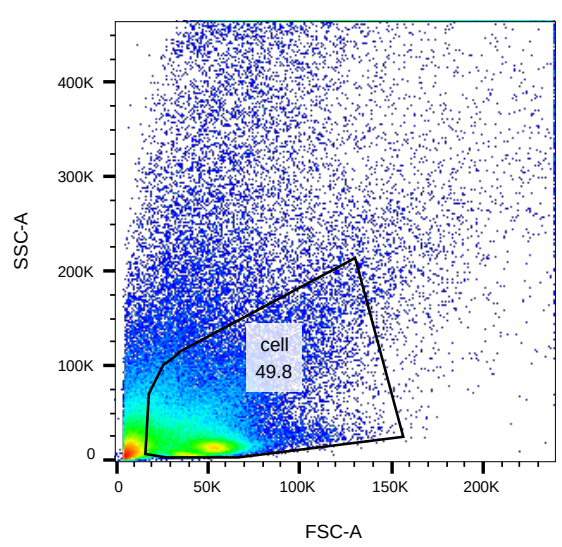

20251121_contra-2_002.fcs
Ungated
130461

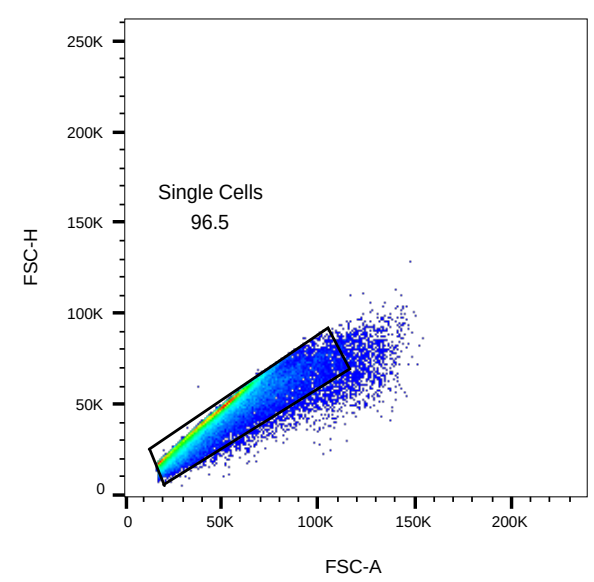

20251121_contra-2_002.fcs
cell
64928

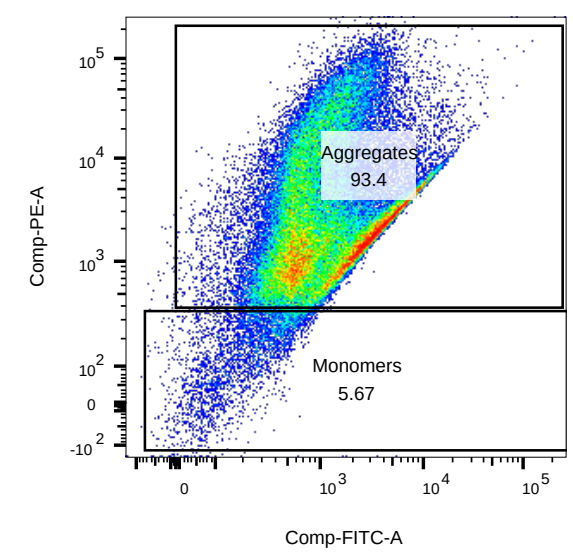

20251121_contra-2_002.fcs
Single Cells
62629
