# Supplementary material for: Exposure-duration-associated auditory dysfunction and cochlear presynaptic ribbon alterations during simulated high-altitude hypobaric hypoxia in mice
Source: Front Neurol. 2026 Oct 15;17:1963563. doi: 10.3389/fneur.2026.1963563 (PMC13716653; doi:10.3389/fneur.2026.1963563)
Supplement: Supplementary file 2 [file Table_1.docx]

**Supplementary Table S1**

**Exploratory pairwise comparisons of auditory structural oxidative and metabolic outcomes**

All six pairwise comparisons among the ambient-pressure control, 3-day, 15-day and 30-day exposure groups are reported for each outcome, stimulus condition or cochlear turn. Mean differences are calculated as group A minus group B. Each animal is one biological replicate. Exposure groups contain different animals assessed at terminal endpoints; these comparisons do not measure recovery within individual animals.

Tukey’s honestly significant difference procedure was used after one-way ANOVA for ATP, CtBP2, 4-HNE and the indicated ABR outcomes. Games–Howell comparisons were used after Welch’s ANOVA for 8-OHdG, the indicated ABR outcomes and logit-transformed JC-1 proportions. The repeated-measures analyses of ABR and CtBP2 remain in the main manuscript.

P values and 95% confidence intervals (CIs) account for all six comparisons among four groups separately for each outcome, stimulus condition, turn or JC-1 fraction. Tukey CIs are simultaneous; Games–Howell simultaneous CIs are approximate. No overall multiplicity adjustment was made across these families. These supplementary analyses are exploratory.

Original control-referenced comparisons are retained in the main manuscript. When those comparisons use Dunnett adjustment, their adjustment family differs from the Tukey family reported here; control-comparison P values and CIs may therefore differ.

(1) **ATP content**

Five mice per group. Mean differences and CIs are expressed in nmol/mg protein. Bilateral cochleae from each mouse were pooled as one sample. Tukey comparisons.

| **Comparison (A−B)** | **Mean difference** | **95% CI** | **Adjusted P** |
| --- | --- | --- | --- |
| 3d − Control | −1.210 | −1.907 to −0.512 | 0.0007 |
| 15d − Control | −5.669 | −6.366 to −4.972 | <0.0001 |
| 30d − Control | −3.193 | −3.890 to −2.496 | <0.0001 |
| 15d − 3d | −4.459 | −5.156 to −3.762 | <0.0001 |
| 30d − 3d | −1.984 | −2.681 to −1.287 | <0.0001 |
| 30d − 15d | 2.475 | 1.778 to 3.173 | <0.0001 |

(2) **CtBP2 puncta per inner hair cell**

Three mice per group. Mean differences and CIs are expressed in puncta per inner hair cell (IHC). Each mouse contributed one animal-level value for each of the three cochlear turns. Each within-turn analysis contains one value per mouse; the three turns are not treated as independent animals. Tukey comparisons.

**Apical turn**

| **Comparison (A−B)** | **Mean difference** | **95% CI** | **Adjusted P** |
| --- | --- | --- | --- |
| 3d − Control | −0.467 | −2.373 to 1.440 | 0.8598 |
| 15d − Control | −2.500 | −4.407 to −0.593 | 0.0128 |
| 30d − Control | −1.167 | −3.073 to 0.740 | 0.2781 |
| 15d − 3d | −2.033 | −3.940 to −0.127 | 0.0371 |
| 30d − 3d | −0.700 | −2.607 to 1.207 | 0.6572 |
| 30d − 15d | 1.333 | −0.573 to 3.240 | 0.1922 |

**Middle turn**

| **Comparison (A−B)** | **Mean difference** | **95% CI** | **Adjusted P** |
| --- | --- | --- | --- |
| 3d − Control | −0.233 | −1.947 to 1.481 | 0.9705 |
| 15d − Control | −3.182 | −4.896 to −1.468 | 0.0015 |
| 30d − Control | −0.333 | −2.047 to 1.381 | 0.9219 |
| 15d − 3d | −2.948 | −4.662 to −1.235 | 0.0025 |
| 30d − 3d | −0.100 | −1.814 to 1.614 | 0.9975 |
| 30d − 15d | 2.848 | 1.135 to 4.562 | 0.0031 |

**Basal turn**

| **Comparison (A−B)** | **Mean difference** | **95% CI** | **Adjusted P** |
| --- | --- | --- | --- |
| 3d − Control | −3.567 | −6.197 to −0.936 | 0.0106 |
| 15d − Control | −3.600 | −6.231 to −0.969 | 0.0101 |
| 30d − Control | −1.767 | −4.397 to 0.864 | 0.2165 |
| 15d − 3d | −0.033 | −2.664 to 2.597 | >0.9999 |
| 30d − 3d | 1.800 | −0.831 to 4.430 | 0.2052 |
| 30d − 15d | 1.833 | −0.797 to 4.464 | 0.1943 |

Tukey adjustment is applied separately to the six comparisons within each turn. These intervals and P values do not control multiplicity jointly across the three turns**.**

(3) **JC1 mitochondrial polarization fractions**

Three mice per group. Bilateral cochleae from each mouse were pooled as one sample. The proportion p was transformed as ln[p/(1−p)], with p equal to the measured percentage divided by 100. Games–Howell comparisons.

Mean differences and CIs below are on the logit scale. They are neither percentage-point differences nor odds ratios. Raw percentages and their variability are presented in the main manuscript.

**Monomer high fraction**

| **Comparison (A−B)** | **Mean difference** | **95% CI** | **Adjusted P** |
| --- | --- | --- | --- |
| 3d − Control | 0.526 | −1.053 to 2.104 | 0.4968 |
| 15d − Control | 0.843 | −1.157 to 2.844 | 0.2385 |
| 30d − Control | 2.651 | 0.824 to 4.477 | 0.0222 |
| 15d − 3d | 0.318 | −0.729 to 1.365 | 0.4027 |
| 30d − 3d | 2.125 | 1.260 to 2.991 | 0.0041 |
| 30d − 15d | 1.807 | 1.336 to 2.279 | 0.0021 |

**Aggregate high fraction**

| **Comparison (A−B)** | **Mean difference** | **95% CI** | **Adjusted P** |
| --- | --- | --- | --- |
| 3d − Control | −0.516 | −2.140 to 1.109 | 0.5202 |
| 15d − Control | −0.831 | −2.899 to 1.237 | 0.2541 |
| 30d − Control | −2.580 | −4.533 to −0.626 | 0.0282 |
| 15d − 3d | −0.316 | −1.385 to 0.754 | 0.4005 |
| 30d − 3d | −2.064 | −2.976 to −1.153 | 0.0068 |
| 30d − 15d | −1.749 | −2.124 to −1.373 | 0.0018 |

Games–Howell adjustment covers six comparisons among four groups separately for each fraction. The monomer-high and aggregate-high fractions derive from the same JC-1 assay and describe related aspects of mitochondrial polarization; they are not independent biological effects or independent replications.

**(4) ABR thresholds**

Ten mice per group. Each mouse contributed one value for each stimulus condition. Mean differences and CIs are expressed in dB SPL. Comparisons are performed separately within each condition; repeated measurements from the same animal are not treated as independent mice. Tukey comparisons.

**Click threshold**

| **Comparison (A−B)** | **Mean difference** | **95% CI** | **Adjusted P** |
| --- | --- | --- | --- |
| 3d − Control | 8.000 | −2.042 to 18.042 | 0.1583 |
| 15d − Control | 31.500 | 21.458 to 41.542 | <0.0001 |
| 30d − Control | 17.000 | 6.958 to 27.042 | 0.0003 |
| 15d − 3d | 23.500 | 13.458 to 33.542 | <0.0001 |
| 30d − 3d | 9.000 | −1.042 to 19.042 | 0.0925 |
| 30d − 15d | −14.500 | −24.542 to −4.458 | 0.0023 |

**4 kHz threshold**

| **Comparison (A−B)** | **Mean difference** | **95% CI** | **Adjusted P** |
| --- | --- | --- | --- |
| 3d − Control | 10.500 | 0.671 to 20.329 | 0.0325 |
| 15d − Control | 29.000 | 19.171 to 38.829 | <0.0001 |
| 30d − Control | 18.000 | 8.171 to 27.829 | 0.0001 |
| 15d − 3d | 18.500 | 8.671 to 28.329 | <0.0001 |
| 30d − 3d | 7.500 | −2.329 to 17.329 | 0.1874 |
| 30d − 15d | −11.000 | −20.829 to −1.171 | 0.0233 |

**8 kHz threshold**

| **Comparison (A−B)** | **Mean difference** | **95% CI** | **Adjusted P** |
| --- | --- | --- | --- |
| 3d − Control | 5.000 | −5.584 to 15.584 | 0.5861 |
| 15d − Control | 29.000 | 18.416 to 39.584 | <0.0001 |
| 30d − Control | 9.000 | −1.584 to 19.584 | 0.1193 |
| 15d − 3d | 24.000 | 13.416 to 34.584 | <0.0001 |
| 30d − 3d | 4.000 | −6.584 to 14.584 | 0.7402 |
| 30d − 15d | −20.000 | −30.584 to −9.416 | <0.0001 |

**ABR wave I latency**

Ten mice per group. Wave I was assessed at 90 dB SPL. Mean differences and CIs are expressed in milliseconds. Each comparison family contains all six group comparisons within the stated frequency.

**4 kHz latency using Tukey comparisons**

| **Comparison (A−B)** | **Mean difference** | **95% CI** | **Adjusted P** |
| --- | --- | --- | --- |
| 3d − Control | 0.282 | 0.038 to 0.526 | 0.0184 |
| 15d − Control | 0.319 | 0.075 to 0.563 | 0.0063 |
| 30d − Control | 0.264 | 0.020 to 0.508 | 0.0300 |
| 15d − 3d | 0.037 | −0.207 to 0.281 | 0.9767 |
| 30d − 3d | −0.018 | −0.262 to 0.226 | 0.9972 |
| 30d − 15d | −0.055 | −0.299 to 0.189 | 0.9294 |

**8 kHz latency using Games Howell comparisons**

| **Comparison (A−B)** | **Mean difference** | **95% CI** | **Adjusted P** |
| --- | --- | --- | --- |
| 3d − Control | 0.417 | 0.164 to 0.670 | 0.0014 |
| 15d − Control | 0.265 | 0.099 to 0.431 | 0.0014 |
| 30d − Control | 0.334 | 0.137 to 0.531 | 0.0008 |
| 15d − 3d | −0.152 | −0.399 to 0.095 | 0.3176 |
| 30d − 3d | −0.083 | −0.347 to 0.181 | 0.8068 |
| 30d − 15d | 0.069 | −0.120 to 0.258 | 0.7285 |

The heteroscedastic procedure was used for 8 kHz latency in accordance with the variance assessment. Non-significant exposure-group comparisons do not establish equivalence or a plateau. Wave I measurements at a fixed sound pressure level are not matched for sensation level when thresholds differ.

**ABR wave I amplitude**

Ten mice per group. Wave I was assessed at 90 dB SPL. Mean differences and CIs are expressed in µV. Each comparison family contains all six group comparisons within the stated frequency.

**4 kHz amplitude using Games Howell comparisons**

| **Comparison (A−B)** | **Mean difference** | **95% CI** | **Adjusted P** |
| --- | --- | --- | --- |
| 3d − Control | −0.611 | −1.541 to 0.319 | 0.2768 |
| 15d − Control | −1.044 | −1.883 to −0.205 | 0.0145 |
| 30d − Control | −0.582 | −1.424 to 0.260 | 0.2208 |
| 15d − 3d | −0.433 | −1.061 to 0.195 | 0.2289 |
| 30d − 3d | 0.029 | −0.603 to 0.661 | 0.9991 |
| 30d − 15d | 0.462 | 0.075 to 0.849 | 0.0163 |

**8 kHz amplitude using Tukey comparisons**

| **Comparison (A−B)** | **Mean difference** | **95% CI** | **Adjusted P** |
| --- | --- | --- | --- |
| 3d − Control | −0.918 | −2.171 to 0.335 | 0.2169 |
| 15d − Control | −2.333 | −3.586 to −1.080 | <0.0001 |
| 30d − Control | −0.053 | −1.306 to 1.200 | 0.9995 |
| 15d − 3d | −1.415 | −2.668 to −0.162 | 0.0217 |
| 30d − 3d | 0.865 | −0.388 to 2.118 | 0.2633 |
| 30d − 15d | 2.280 | 1.027 to 3.533 | 0.0001 |

The heteroscedastic procedure was used for 4 kHz amplitude in accordance with the variance assessment. Higher amplitudes in one terminal exposure group do not demonstrate recovery within individual animals

**Sensitivity of the 4 kHz threshold comparison**

For the 30-day minus 15-day contrast, the mean difference was −11.000 dB SPL. Tukey’s procedure gave a 95% CI of −20.829 to −1.171 and adjusted P = 0.0233. A heteroscedastic Games–Howell sensitivity analysis of the same four-group family gave an approximate 95% CI of −23.020 to 1.020 and adjusted P = 0.0778. This contrast is sensitive to the variance model and should not be interpreted as robust evidence of threshold recovery.

**(5) Oxidative stress marker fluorescence**

Five mice per group. Each observation is an animal-level mean fluorescence measurement. Analyses use background-corrected values before normalization to the control-group mean; mean differences and CIs are expressed in arbitrary fluorescence units. The main figure displays relative fluorescence values.

**4 HNE using Tukey comparisons**

| **Comparison (A−B)** | **Mean difference** | **95% CI** | **Adjusted P** |
| --- | --- | --- | --- |
| 3d − Control | 41.705 | 17.981 to 65.430 | 0.0006 |
| 15d − Control | 83.824 | 60.100 to 107.548 | <0.0001 |
| 30d − Control | 49.901 | 26.176 to 73.625 | <0.0001 |
| 15d − 3d | 42.119 | 18.394 to 65.843 | 0.0006 |
| 30d − 3d | 8.195 | −15.529 to 31.920 | 0.7580 |
| 30d − 15d | −33.923 | −57.648 to −10.199 | 0.0043 |

**8 OHdG using Games Howell comparisons**

| **Comparison (A−B)** | **Mean difference** | **95% CI** | **Adjusted P** |
| --- | --- | --- | --- |
| 3d − Control | 51.091 | 20.989 to 81.193 | 0.0057 |
| 15d − Control | 38.309 | 13.569 to 63.050 | 0.0078 |
| 30d − Control | −1.299 | −12.809 to 10.211 | 0.9822 |
| 15d − 3d | −12.782 | −44.972 to 19.408 | 0.5985 |
| 30d − 3d | −52.390 | −82.722 to −22.058 | 0.0059 |
| 30d − 15d | −39.608 | −64.485 to −14.732 | 0.0076 |

The Games–Howell procedure accounts for unequal group variances in 8-OHdG fluorescence. Comparisons of exposure groups quantify cross-sectional differences in marker fluorescence, not recovery within individual animals. Absence of a significant 30-day versus control difference does not establish equivalence to control.
