# Supplementary material for: Exposure-duration-associated auditory dysfunction and cochlear presynaptic ribbon alterations during simulated high-altitude hypobaric hypoxia in mice
Source: Front Neurol. 2026 Oct 15;17:1963563. doi: 10.3389/fneur.2026.1963563 (PMC13716653; doi:10.3389/fneur.2026.1963563)
Supplement: Supplementary file 3 [file Table_2.docx]

**Supplementary Table S2**

**Allocation of animals and cochleae to terminal assessments and tissue assays within each experimental group**

Counts are per experimental group: 20 mice and 40 cochleae. The same allocation applied to the ambient-pressure control and the nominal 3-, 15-, and 30-day exposure groups (80 mice in total).

| **Terminal subgroup** | **Mice** | **Cochlear use** | **Cochleae assayed** | **Cochleae stored** |
| --- | --- | --- | --- | --- |
| ABR | 3 | One randomly selected ear for OHC and CtBP2 analysis on the same whole mount; opposite ear stored. | 3 | 3 |
| ABR | 5 | One randomly selected ear for cryosections and separate 4-HNE and 8-OHdG-related staining; opposite ear stored. | 5 | 5 |
| ABR | 2 | Both cochleae stored. | 0 | 4 |
| Non-ABR blood collection | 5 | Both cochleae pooled within each mouse for ATP measurement. | 10 | 0 |
| Non-ABR blood collection | 3 | Both cochleae pooled within each mouse for JC-1 analysis. | 6 | 0 |
| Non-ABR blood collection | 2 | Both cochleae stored. | 0 | 4 |
| **Total** | **20** | **All 40 cochleae accounted for.** | **24** | **16** |

Rows represent mutually exclusive sets of animals. All 10 mice in the ABR subgroup underwent left-ear ABR recording. All 10 mice in the non-ABR subgroup underwent terminal blood collection without prior ABR testing. “Cochleae assayed” refers to the reported tissue assays and excludes ABR recordings.

At tissue collection, three mice were randomly selected from the ABR subgroup for whole-mount analyses. Five different ABR-tested mice provided cryosections. One ear was randomly selected for histology in each of these eight mice. OHC and CtBP2 measurements used the same cochlea and preparation; oxidative markers were evaluated on separate cryosections. The histological ear was not necessarily the ABR-tested left ear.

ATP and JC-1 assays used different mice. For each assay, the two cochleae from one mouse were pooled into one biological sample; samples were not pooled across mice. Thus, ATP n = 5 and JC-1 n = 3 per group, rather than 10 and 6 independent samples.

Stored material comprised 16 cochleae from 12 mice per group: eight contralateral cochleae from histology animals and both cochleae from four other mice. All were stored at −80°C. These four mice contributed ABR or hematological measurements; only their cochleae were reserved for future assays. The four technical-pilot mice were separate from the 80 formal-study mice and are not included in this table.

Abbreviations: ABR, auditory brainstem response; OHC, outer hair cell; CtBP2, C-terminal binding protein 2; ATP, adenosine triphosphate; 4-HNE, 4-hydroxynonenal; 8-OHdG, 8-hydroxy-2′-deoxyguanosine. The 15A3 endpoint is described as 8-OHdG-related immunoreactivity because the antibody also recognizes other oxidized guanine species.
